# Chemical Constituents and Antibacterial Properties of *Indocalamus latifolius* McClure Leaves, the Packaging Material for “Zongzi”

**DOI:** 10.3390/molecules200915686

**Published:** 2015-08-28

**Authors:** Jia Sun, Hang Xun, Jin Yu, Feng Tang, Yong-De Yue, Xue-Feng Guo

**Affiliations:** State Forestry Administration Key Open Laboratory, International Centre for Bamboo and Rattan, Beijing 100102, China; E-Mails: sunjia@icbr.ac.cn (J.S.); xunhang@icbr.ac.cn (H.X.); ahyujin@163.com (J.Y.); Fengtang@icbr.ac.cn (F.T.); gxf61622@icbr.ac.cn (X.-F.G.)

**Keywords:** indocalamus latifolius mcclure, leaf extract, norsesquiterpenoid, 8-4′-oxyneolignan, packaging material, antibacterial property

## Abstract

The glutinous rice dumpling named “Zongzi” in Chinese is a type of traditional food that is popular in East Asian countries. “Zongzi” is made of glutinous rice and wrapped in the leaves of *Indocalamus latifolius* McClure as the packaging material. Four new compounds, latifoliusine A (**2**), (7*S*,8*R*) syringylglycerol-8-*O*-4′-sinapyl ether 4-*O*-β-d-glucopyranoside (**7**), (7*S*,8*S*) syringylglycerol-8-*O*-4′-sinapyl ether 7-*O*-β-d-glucopyranoside (**8**), and (7*R*,8*S*) syringylglycerol-8-*O*-4′-sinapyl ether 7-*O*-β-d-glucopyranoside (**10**), along with six known compounds (**1**, **3**–**6** and **9**) were isolated from *I. latifolius* McClure leaves. The structures and relative configurations of the compounds were determined by detailed spectroscopic analysis, high-resolution electrospray ionization mass spectroscopy (HRESIMS), heteronuclear single quantum correlation (HSQC), heteronuclear multiple bond correlation (HMBC), nuclear overhauser enhancement (NOE) and circular dichroism (CD). All of the isolated compounds were screened for their antibacterial activities *in vitro*. The results indicated that apigenin 6-*C*-α-l-arabinopyranosyl-8-*C*-β-d-glucopyranoside (**5**) and apigenin 7-*O*,8-*C*-di-glucopyranoside (**6**) have antibacterial activities against four bacterial strains (*Staphylococcus aureus*, *Bacillus thuringiensis*, *Escherichia coli* and *Pseudomonas solanacearum*).

## 1. Introduction

“Zongzi”, which is believed to have a history of more than 2000 years, is a type of famous Chinese food that is also popular in many Asian countries [1]. It is made of glutinous rice and wrapped in the large flat leaves of *Indocalamus latifolius* McClure. “Zongzi” has been characterized by a long shelf life since ancient times.

*Indocalamus latifolius* McClure is widely distributed and cultivated in Southern China [2]. It belongs to the same genus as *Indocalamus nakai*, which is reported to have polysaccharides [3,4], metal elements [1], flavonoids [5,6], and volatile components [7] in its leaf extracts and possess anticancer, antitumor, and antioxidative effects, as well as antibacterial activity [8,9].

In our previous research, several new compounds have been identified from the leaves of different bamboo species including the following: Three novel lignans were isolated from *Bambusa* *tuldoides* Munro [10]; a new polyketide derivative named Amarusine A was isolated from the leaves of *Pleioblastus amarus* [11]; two new compounds, xylitol 1-*O*-(6′-*O*-*p*-hydroxylbenzoyl)-glucopyranoside and bambulignan B, were isolated from the leaves of *Pleioblastus amarus* (Keng) keng f [12]; and four diastereoisomeric oxyneolignans were isolated and characterized from *Bambusa tuldoides* Munro [13]. In the present research, on the basis of our continuing research interest in the phytochemistry of bamboo, we examined the phytoconstituents of *I. latifolius* McClure leaves in detail and their antibacterial activities against two Gram-positive and two Gram-negative bacterial strains for the first time.

## 2. Results and Discussion

### 2.1. Structural Elucidation

Repeated chromatography over Sephadex LH-20, macroporous resin and Rp-18 columns as well as preparative HPLC of the 95% ethanol extract from *I. latifolius* McClure leaves led to the isolation of four new compounds, latifoliusine A (**2**), (7*S*,8*R*) syringylglycerol-8-*O*-4′-sinapyl ether 4-*O*-β-d-glucopyranoside (**7**), (7*S*,8*S*) syringylglycerol-8-*O*-4′-sinapyl ether 7-*O-*β-d-glucopyranoside (**8**) and (7*R*,8*S*) syringylglycerol-8-*O*-4′-sinapyl ether 7-*O-*β-d-glucopyranoside (**10**) along with six known compounds.

**Figure 1:**
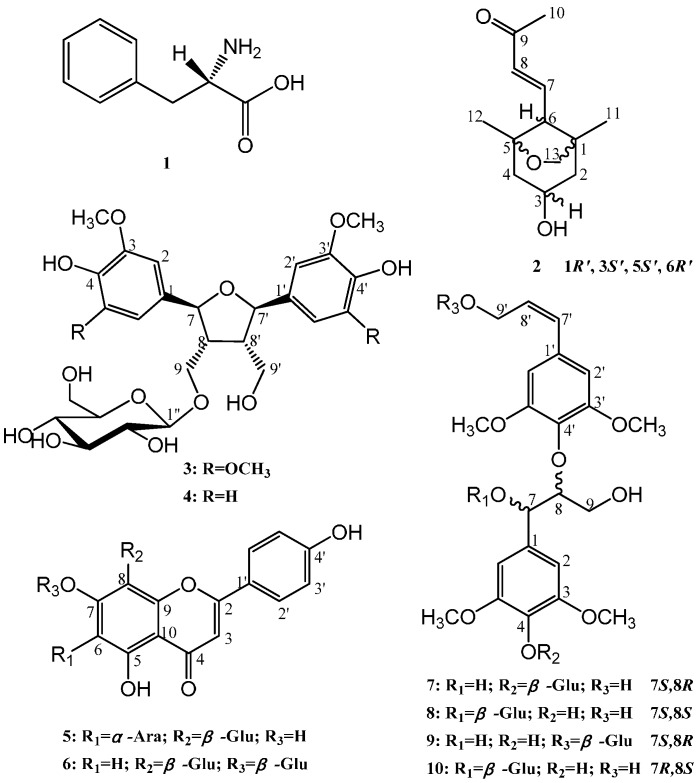
Compounds **1**–**10** isolated from *Indocalamus latifolius* McClure leaves.

The six known compounds were identified (Figure 1) as L-phenylalanine (**1**) [14], dihydroxymethyl-bis(3,5-dimethoxy-4-hydroxyphenyl) tetrahydrofuran-9-*O*-β-d-glucopyranoside (**3**) [15], *rel*-(7*R*,8*S*,7′*S*,8′*R*)-4,9,4′,9′-tetrahydroxy-3,3′-dimethoxy-7,7′-epoxylignan 9-*O-*β-d-glucopyranoside (**4**) [16], apigenin 6-*C-*α-l-arabinopyranosyl-8-*C-*β-d-glucopyranoside (**5**) [17], apigenin 7-*O*,8-*C*-*di*-glucopyranoside (**6**) [18], and (*7S*,*8S*) syringylglycerol-8-*O*-4′-sinapyl ether 9′-*O-*β-d-glucopyranoside (**9**) [19] through comparing their spectroscopic and physical data with those of previous reports.

**Figure 2:**
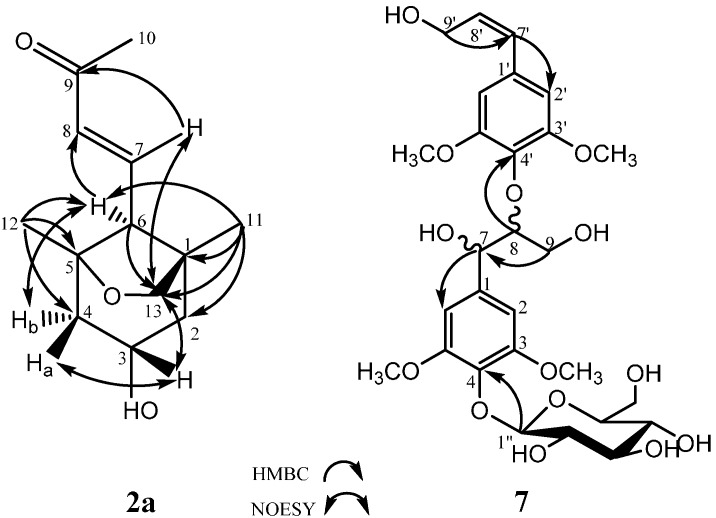
Significant HMBC and NOESY correlations of compounds **2a** and **7**.

**Figure 3:**
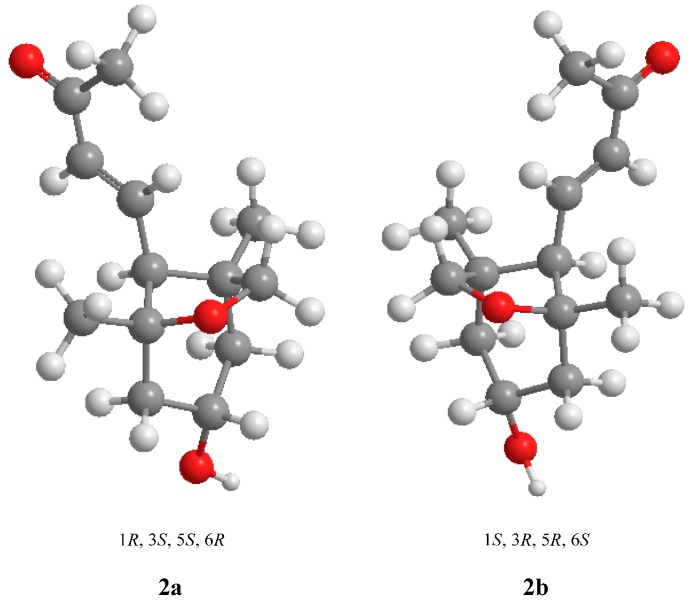
The relative configuration of compound **2**.

New compound **2** was purified as a yellowish oil ([α]_D_ = +36.1°; *c* = 0.70, methanol), and its molecular formula, C_13_H_20_O_3_, was determined by positive HRESIMS (*m*/*z* 247.1313 [M + Na]^+^, calculated 247.1310) and suggests four degrees of unsaturation. The IR spectrum showed characteristic hydroxyl (3424 cm^−1^), methylene (2928 cm^−1^) and double bond (1670 cm^−1^) absorption bands. The ^1^H-NMR spectrum indicated the presence of one *trans*-double bond, as supported by hydrogen signals at δ_H_ 6.67 (1H, *dd*, *J* = 16.0, 11.0) and δ_H_ 6.12 (1H, *d*, *J* = 16.0). Additionally, one oxymethine at δ_H_ 3.91 (1H, *m*); one oxymethylene at δ_H_ 3.64 (2H, *m*); two methylenes at δ_Ha_ 1.87 and δ_Hb_ 1.31 (1H, *dd*, *J* = 12.5, 6.0) and at δ_Ha_ 1.94 and δ_Hb_ 1.36 (1H, *dd*, *J* = 12.5, 6.5); and three methylenes at δ_H_ 2.24 (3H, *s*), δ_H_ 1.02 (3H, *s*) and δ_H_ 0.85 (3H, *s*) were observed in the ^1^H-NMR spectrum, as well as active hydrogen signals at δ_H_ 4.58 (1H, *s*). The ^13^C-NMR spectrum revealed the resonances of thirteen carbons. Based on the DEPT spectrum, these resonances included one ketone carbon at δ_C_ 198.1; two olefinic carbons at δ_C_ 134.1 and δ_C_ 146.1; two quaternary carbons at δ_C_ 83.8 and δ_C_ 45.1; two methines at δ_C_ 64.7 and δ_C_ 60.6; three methylenes at δ_C_ 75.9, δ_C_ 48.7 and δ_C_ 47.8; and three methyls at δ_C_ 27.2, δ_C_ 23.9 and δ_C_ 20.6. Using the analysis of the degree of unsaturation, these data indicated that compound **2** contained two rings. In the HMBC spectrum (Figure 2), correlations of δ 0.85 (H-11) with δ_C_ 45.1 (C-1), δ_C_ 47.8 (C-2), δ_C_ 60.6 (C-6) and δ_C_ 75.9 (C-13), and δ_C_ 1.02 (H-12) with δ_C_ 48.7 (C-4), δ_C_ 83.8 (C-5) and δ_C_ 60.6 (C-6) were observed, suggesting that δ_C_ 45.1 (C-1) and δ_C_ 83.8 (C-5) were the bridgehead carbons of a furan ring (C-13, C-1, C-6 and C-5) and a six-membered ring (C-1, C-2, C-3, C-4, C-5 and C-6), respectively. Furthermore, the protons of the double-bond, δ_H_ 6.12 (H-7) and δ_H_ 6.67 (H-8), were correlated to δ_H_ 60.6 (C-6) and δ_H_ 198.1 (C-9), which confirmed that the butenone group was linked to C-6 (Figure 2). The relative configuration was further determined by NOESY correlations between δ 3.91 (H-3) and both δ_H_ 3.64 (H-13) and δ_H_ 1.94 (H-4a) and between δ_H_ 2.29 (H-6) and δ_H_ 1.36 (H-4b), confirming that H-3 and H-13 were on the same side of the molecule and that H-6 was positioned on the other side of the molecule (Figure 2). Based on these data, the pair of enantiomers of 1*R*′,3*S*′,5*S*′,6*R*′ (**2a**: 1*R*,3*S*,5*S*,6*R* and **2b**: 1*S*,3*R*,5*R*,6*S*) was determined to be the relative configuration for compound **2** (Figure 3). Thus, the structure of compound **2** was elucidated as depicted and named latifoliusine A (Figure 1).

New compound **7** was obtained as a yellow amorphous powder ([α]_D_ = −15.9°; *c* = 1.0, methanol). Its molecular formula, C_28_H_38_O_14_, was established by negative HRESIMS (*m*/*z* 597.2179 [M − H]^−^, calculated 597.2183). The IR spectrum showed absorption bands characteristic of hydroxyl groups (3381 cm^−1^), methylenes (2927 cm^−1^) and aromatic rings (1654 and 1451 cm^−1^). The ^1^H NMR spectrum exhibited signals for two 3,5-dimethoxy-4-hydroxyphenyl moieties, which included two aromatic hydrogen signals at δ_H_ 6.74 (2H, *s*) and δ_H_ 6.70 (2H, *s*); four methoxyl groups at δ_H_ 3.73 (6H, *s*) and δ_H_ 3.72 (6H, *s*); one *trans* double bond at δ_H_ 6.47 (1H, *d*, *J* = 16.0) and δ_H_ 6.34 (1H, *dt*, *J* = 16.0, 5.0); and one anomeric proton at δ_H_ 4.86 (1H, *d*, *J* = 7.5), indicating a β-glycosidic linkage for the d-glucose [10,20,21]. Moreover, there were other alkyl groups and signals attributed to a β-d-glucopyranosyl unit. The ^13^C-NMR spectra showed carbon signals corresponding to the chemical units described above and confirmed their presence. Furthermore, two oxymethines at δ_C_ 71.1 and δ_C_ 86.7 and an oxymethylene at δ_C_ 60.5 were attributed to an arylglyceryloxy unit. These spectral features indicated that compound **7** was an 8-*O*-4′-type neolignan glycoside formed by two phenylpropanoid glycosides. In the HMBC spectrum, the correlation between δ_H_ 4.09 (H-8) and δ_C_ 135.8 (C-4′) confirmed the neolignan structures, and the correlation between δ_H_ 4.86 (H-1′′) and δ_C_ 138.4 (C-4) implied that the *O*-glycoside was linked to δ_C_ 138.4 (C-4) (Figure 2). The *erythro-* configuration of **7** at C-7 and C-8 was determined by the *J*_7,8_-value (4.0 Hz) in the ^1^H-NMR spectrum [22,23]. The absolute configuration at C-7 and C-8 of compound **7** was assigned to be (*7S*,*8R*) based on the negative Cotton effect (*∆*ε_243.5nm_ = −0.4727) in the CD spectrum [24,25]. Therefore, the structure of **7** was determined to be (*7S,8R*) syringylglycerol-8-*O*-4′-sinapyl ether 4-*O*-β-d-glucopyranoside (Figure 1).

New compound **8** was obtained as a yellow powder ([α]_D_ = −3.11°; *c* = 1.0, methanol), and its molecular formula was determined to be C_28_H_38_O_14_ by negative HRESIMS (*m*/*z* 633.1952 [M + Cl]^−^, calculated 633.1950). The IR spectrum showed absorption bands characteristic for hydroxyl groups (3385 cm^−1^), methylenes (2927 cm^−1^) and aromatic rings (1584 and 1460 cm^−1^). The ^1^H NMR and ^13^C-NMR spectra of compound **8** also exhibited signals characteristic of an 8-*O*-4′-type neolignan glycoside. In the HSQC spectrum, an anomeric proton signal at δ_H_ 4.35 (1H, *d*, *J* = 7.5) correlated with a corresponding carbon signal at δ_C_ 102.6 in the ^1^H- and ^13^C-NMR spectra, respectively, which suggested that compound **8** had one terminal β-d-glucopyranosyl unit. In the HMBC analysis, the presence of a cross-peak between the anomeric proton at δ_H_ 4.35 (H-1″) and δ_C_ 79.0 (C-7) revealed the location of the glucosidic linkage at the C-7 position. In the ^1^H- and ^13^C-NMR spectra (Table 1), the signals of **8** were similar to the planar structure of 3-hydroxy-1-(4-hydroxy-3,5-dimethoxyphenyl)-2-[4-(3-hydroxy-1-(*E*)-propenyl)-2,6-dimethoxyphenoxy]propyl-β-d-glucopyranoside [26]. However, the *J*_7,8_ value was not directly applicable to distinguish the *erythro-* and *threo-* forms of 8-4′-oxyneolignan arylglycerol 7-*O*-β-d-glucopyranosides [13,27]. Therefore, the configuration of **8** will be further elucidated with the description of compound **10**.

New compound **10** was also obtained as a yellow powder ([α]_D_ = +4.55°; *c* = 1.0, methanol). The molecular formula of **10**, C_28_H_38_O_14_, was confirmed by negative HRESIMS (*m*/*z* 633.1953 [M + Cl]^−^, calculated 633.1950) and coincided with that of **8**. The IR and UV spectra of **8** and **10** showed similar absorption patterns. The ^1^H- and ^13^C-NMR spectra of **10** were very similar to those of **8**, suggesting that the overall structure of **10** was the same as that of **8**. Moreover, the HSQC and HMBC correlations of **10** corroborated the aforementioned deduction.

By comparing the ^1^H- and ^13^C-NMR spectral data of **8** and **10**, there were several differences in the field shifts for C-1, C-7, C-8, and C-9 that could be observed (Table 1), which indicated compound **8** and **10** were chiral isomers at C-7 and C-8. The chemical shift difference between C-8 and C-7 (Δδ_C8–C7_) can distinguish the *erythro-* and *threo-*isomers. In DMSO-*d*_6_, the Δδ_C8–C7_ value of the *threo-*glycoside was larger than that of the *erythro*-glycoside by approximately 1 ppm [28,29,30]. Therefore, the Δδ_C8–C7_ value of the *threo-*glycoside **8** (5.6 ppm) was larger than that of the *erythro-* isomer **10** (4.2 ppm). Furthermore, the positive Cotton effect in the CD spectra of **8** (∆ε_237.5nm_ = +2.0655) and **10** (∆ε_231nm_ = +1.6611) indicated that the absolute configuration of **8** was (7*S*,8*S*) and that **10** was (7*R*,8*S*). Consequently, the structures of compounds **8** and **10** were determined to be (7*S*,8*S*) syringylglycerol-8-*O*-4′-sinapyl ether 7-*O*-β-d-glucopyranoside and (7*R*,8*S*) syringylglycerol-8-*O*-4′-sinapyl ether 7-*O*-β-d-glucopyranoside, respectively (Figure 1).

Detailed ^1^H, ^13^C, HSQC, HMBC, NOESY, IR, HRESIMS and UV spectra of compound (**2**) as well as ^1^H, ^13^C, HSQC, HMBC IR, HRESIMS, CD, and UV spectra of compounds **7**, **8**, and **10** are provided in the supplementary data.

**Table 1:** NMR spectroscopic data (measured at 500 MHz) of the isolated compounds **2**, **7**, **8**, and **10** in DMSO from the leaves of *Indocalamus latifolius* McClure.

	Compound 2			Compound 7		Compound 8		Compound 10	
No.	δ_C_	δ_H_, *J* in Hz	No.	δ_C_	δ_H_, *J* in Hz	δ_C_	δ_H_, *J* in Hz	δ_C_	δ_H_, *J* in Hz
1	45.1		1	133.9		129.4		130.3	
2	47.8	1.87, 1H, *dd*, 12.5, 6.0 (Ha) 1.31, 1H, *dd*, 12.5, 6.0 (Hb)	2	105.5	6.74, 1H, *s*	105.7	6.73, 1H, *s*	106.3	6.70, 1H, *s*
			3	152.3		147.8		147.7	
3	64.7	3.91, 1H, *m*	4	138.4		135.2		134.6	
4	48.7	1.94, 1H, *dd*, 12.5, 6.5 (Ha) 1.36, 1H, *dd*, 12.5, 6.5 (Hb)	5	152.3		147.8		147.7	
			6	105.5	6.74, 1H, *s*	105.7	6.73, 1H, *s*	106.3	6.70, 1H, *s*
5	83.8		7	71.7	4.87, 1H, *d*, 4.0	79.0	5.12, 1H, *d*, 4.0	79.7	4.98, 1H, *d*, 6.5
6	60.6	2.29, 1H, *d*, 11.0	8	86.7	4.09, 1H, *m*	84.6	4.25, 1H, *m*	83.9	4.07, *m*
7	146.1	6.67, 1H, *dd*, 16.0, 11.0	9	60.5	3.66, 3.28, 2H, *m*	60.7	3.61, 3.20, 1H, *m*	59.8	3.57, 3.15, 2H, *m*
8	134.1	6.12, 1H, *d*, 16.0	1′	132.8		135.9		135.2	
9	198.1		2′	104.1	6.70, 1H, *s*	104.1	6.75, 1H, *s*	104.0	6.70, 1H, *s*
10	27.2	2.24, 3H, *s*	3′	153.1		153.2		153.1	
11	20.6	0.85, 3H, *s*	4′	135.8		133.1		132.8	
12	23.9	1.02, 3H, *s*	5′	153.1		153.2		153.1	
13	75.9	3.64, 2H, *m*	6′	104.1	6.70, 1H, *s*	104.1	6.75, 1H, *s*	104.0	6.70, 1H, *s*
3-OH		4.58, 1H, *s*	7′	128.9	6.47, 1H, *d*, 16.0	129.0	6.50,1H, *d*, 16.0	129.0	6.47, 1H, *d*, 16.0
			8′	130.6	6.34, 1H, *dt*, 16.0, 5.0	130.8	6.37, 1H, *dt*, 16.0, 5.0	130.6	6.34, 1H, *dt*, 16. 0, 5.0
			9′	61.8	4.10, 2H, *m*	62.0	4.11, 2H, *m*	61.9	4.10, 2H, *m*
			3,5-OCH_3_ 56.4		3.73, 6H, *s*	56.5	3.78, 6H, *s*	56.3	3.75, 6H, *s*
			3′,5′-OCH_3_ 56.8		3.72, 6H, *s*	56.5	3.74, 6H, *s*	56.4	3.72, 6H, *s*
			4-*O*-glucose			7′-*O*-glucose		7′-*O*-glucose	
			1′′	103.4	4.86, 1H, *d*, 7.5	102.6	4.35, 1H, *d*, 7.5	103.2	4.55, 1H, *d*, 8.0
			2′′	74.6	3.20, 1H, *m*	74.6	3.08, 1H, *m*	74.5	3.07, 1H, *m*
			3′′	76.9	3.20, 1H, *m*	77.8	3.07,1H, *m*	77.4	3.02, 1H, *m*
			4′′	70.4	3.16, 1H, *m*	70.4	3.04, 1H, *m*	70.7	2.99, 1H, *m*
			5′′	77.5	3.03, 1H, *m*	76.9	3.15, 1H, *m*	77.2	3.15, 1H, *m*
			6′′	61.3	3.60, 3.43, 2H, *m*	61.4	3.61, 3.42, 2H, *m*	61.7	3.64, 3.38, 2H, *m*

### 2.2. Antibacterial Activities of the Isolated Compounds

The agar-disk diffusion method is a traditional method for measuring the antibacterial activities of compounds, and their antibacterial effects can be visually observed [31,32,33].

**Figure 4:**
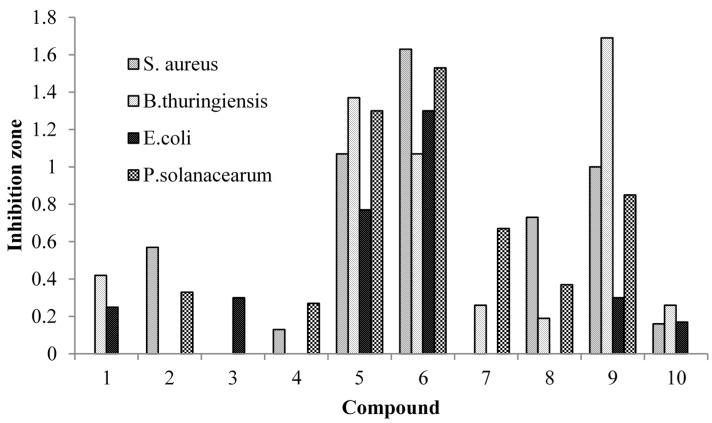
Antibacterial activities of the compounds isolated from the leaves of *Indocalamus latifolius* McClure.

The results of the antibacterial activity tests indicated that the 10 compounds had selective antibacterial properties. Figure 4 shows the zones of inhibition for each compound against the four test strains. All 10 compounds showed inhibition zones, which varied from 0.13 to 1.69 mm.

Compounds **5** and **6** had antibacterial activities against all four bacterial strains and, more notably, these two compounds showed strong antibacterial activities against *S. aureus* and *E. coli*, which are food-contaminating bacteria. Of the remaining two test strains, *B. thuringiensis* was most sensitive to compound **9**, and *P. solanacearum* was most sensitive to compound **6**.

### 2.3. Discussion

Consumers, nowadays, have a strong demand for greener food preservation techniques; hence there is great potential for developing naturally-derived antimicrobial agents. Extensive research has documented that compounds isolated from plants contain a large number of secondary metabolites and possess the capacity to inhibit the growth of bacteria and fungi [34]. The antimicrobial compounds in plants are a part of the self-defense mechanisms for combating harmful microbes in a natural environment [35]. Many of these compounds are under investigation and are not yet exploited commercially. Hao *et al.* [36] found the alcohol extracts of angelica root, banana purée, bay, caraway seed, carrot root, clove (eugenol), marjoram, pimento leaf, and thyme showed inhibition of *A. hydrophila* and *L. monocytogenes* in refrigerated poultry. Ahn *et al.* [37] also found grape seed extract and pine bark extract could control the growth of microorganisms in cooked beef. Kotzekidou *et al.* [38] tested plant extracts and essential oils with potent antimicrobial activities in chocolate at different temperatures and in dry or humidified environment, the most inhibitory action was observed by lemon flavor applied on chocolate inoculated with *E. coli* cocktail culture after storage at 20 °C for 9 days. Martinez-Romero *et al.* [39] reported that the vapor atmosphere of carvacrol could reduce the fungal growth in grape berries.

Another application of natural derived antimicrobials is in the bioactive packaging technologies for food preservation. Seydim [40] found the antimicrobial activity of some spice extracts could be expressed in a whey protein isolate (WPI)-based edible film; hence, they may act as releasable antimicrobial constituents in food packaging. Oussalah [41] studied milk protein-based edible films containing plant essential oils mix on beef muscle slices for controlling the growth of pathogenic bacteria during storage at 4 °C; the film containing oregano showed the most effective against two test bacteria. Nicholson [42] suggested naturally-occurring bio-preservatives could be applied in the food packaging system as part of a multiple hurdle technique, and should lead to increases in both the food safety and shelf-life of perishable foods.

Naturally-derived preservatives for food have been investigated for practical applications in the last 10 years; however, there are also challenges. Plant extracts, especially the EOs, always have strong odor/flavor and may transfer into the food. In this research, we investigated the compounds from a traditional natural packaging material, the leaves of *Indocalamus latifolius* McClure. In addition to the antibacterial capacity, we also found compound (**2**) has a pleasant smell. Thus, the isolated compounds in our research could act as an antimicrobial agent or as a component in antimicrobial packages, and also as an odor/flavor enhancer for packaged foods.

Whereas, the results and data obtained from laboratory *in vitro* experiments may not be applied to food products as foods are complex, the natural antimicrobial agents may offer exclusive advantages for food preservation, and the applications of naturally-derived antimicrobial agents in food will rise steadily in the future.

## 3. Experimental Section

### 3.1. Plant Material

*I. latifolius* McClure leaves were collected from the Century Garden of Bamboos in Yibin city, Sichuan, China. A voucher specimen was deposited in the State Forestry Administration Key Open Laboratory at the International Centre for Bamboo and Rattan in Beijing 100102, China.

### 3.2. Instrumental Equipment

Preparative HPLC was performed on a Shimadzu LC-6AD with an SPD-20A detector (Shimadzu, Kyoto, Japan) using a YMC-Pack ODS-A column (250 mm × 20 mm, 5 μm, YMC, Kyoto, Japan). HPLC-PAD analysis was performed using a Waters 2695-2996 system and a 2996 PDA detector (Waters, Milford, MA, USA) with a YMC-Pack ODS-AQ C_18_ column (250 mm × 4.6 mm, 5 μm, YMC, Kyoto, Japan). IR spectra were collected on a Thermo Nicolet FT-IR NEXUS 670 spectrophotometer (Thermo, Waltham, MA, USA) using KBr pellets, and NMR spectra were collected on Bruker 500 MHz spectrometers (Bruker, Zurich, Switzerland). HRESIMS spectra were obtained with an Agilent 6540 high resolution time-of-flight (Q-TOF) mass spectrometer (Agilent, Santa Clara, CA, USA). Circular dichroism (CD) spectra were recorded in methanol solutions using a JASCO J-815 CD spectrometer (JASCO, Tokyo, Japan). Antibacterial properties were determined by the filter agar-disk diffusion method [34].

### 3.3. Chemicals and Reagents

Column chromatography was performed with macroporous resin (Diaion HP-20, Mitsubishi Chemical Corp., Tokyo, Japan), Rp-18 (50 μm, YMC, Kyoto, Japan) and Sephadex LH-20 (Pharmacia Fine Chemicals, Uppsala, Sweden). All of the reagents and the nutrient agar were purchased from Beijing Chemical Works (Beijing, China) unless otherwise specified. HPLC-grade methanol (MeOH) and ethanol (EtOH) were purchased from Fisher Scientific (Pittsburgh, PA, USA).

### 3.4. Analytical Methods

HPLC analysis utilized a binary elution system consisting of solvent A (MeOH) and solvent B (water containing 0.2% acetic acid) with a YMC-PACK ODS-AQ C_18_ column. The flow rate was 1 mL/min, the column temperature was 30 °C and the injection volume was 10 μL. The PAD detection wavelength monitoring range was 210 to 400 nm. LC-MS analysis was performed to confirm the molecular weights of the compounds using an Rp-18 column and ESI-MS. The mobile phases were solvent A (MeOH) and solvent C (water containing 0.1% formic acid). The flow rate was 0.3 mL/min, the column temperature was 30 °C, and the effluent was monitored at either 220 or 270 nm. The ^1^H-, ^13^C-NMR, and 2D (HSQC, HMBC, and NOE) NMR spectra were recorded on 500 MHz Bruker spectrometers using DMSO-*d*_6_ as the solvent and tetramethylsilane (TMS) as the internal standard. The chemical shifts were expressed in δ (ppm), and the coupling constants were reported in Hertz. The concentration of the compound was 12 mg/mL, the NMR acquisition duration was 2 min for ^1^H-NMR and 5 h for ^13^C-NMR, and the widths of the NMR spectra were 0–14 ppm for ^1^H-NMR and 0–220 ppm for ^13^C-NMR.

### 3.5. Extraction, Isolation, and Purification of the Compounds from *Indocalamus Latifolius* McClure

Dried *I. latifolius* McClure leaves (7 kg) were extracted with 10 L of 95% aqueous ethanol for 24 h at room temperature three times. The solvent was removed under vacuum to collect the filtrates. The concentrated aqueous fraction was separated on a macroporous resin column using a step-wise gradient of water/ethanol (100:0, 85:15, 70:30, 50:50, 30:70, and 5:95) to yield six fractions. Medium-scale preparative performance liquid chromatography was applied to the 30% ethanol fraction (9.6 g) using a Rp-18 column, which was eluted using a step-wise water/methanol gradient (100:0, 80:20, 75:25, 70:30, 65:35, 60:40, 55:45, 50:50, 40:60, 30:70, and 5:95) to yield eleven fractions (1–11). Fraction 3 (128.4 mg) underwent additional column chromatography (CC) over Sephadex LH-20 with water elution, and preparative HPLC was performed with a methanol-water (15:85) elution, which yielded compound **1** (8.5 mg). Fraction 6 (286.3 mg), subjected to the same CC system with methanol/water (25:75) as the elution solvent, yielded compounds **2** (6.8 mg), **3** (52.6 mg) and **4** (27.9 mg). Fraction 7 (168.5 mg) was also subjected to the same two-step CC system and elution with methanol/water (30:70) yielded compounds **5** (12 mg) and **6** (5.8 mg). The two-step CC method was also performed on Fraction 8 (137.6 mg), and the methanol/water ratio used for elution was 35:65, yielding compounds **7** (8.7 mg), **8** (11.2 mg) and **9** (18.2 mg). The same CC was applied to Fraction 9 (320.4 mg), and the HPLC sample was eluted with methanol/water (40:60). This fraction was further precipitated with water, yielding compound **10** (79.0 mg).

*L-phenylalanine* (**1**). White amorphous powder; [α]_D_ = −33.9° (*c* = 0.50, methanol). HRESIMS: C_9_H_11_NO_2_, *m/z* 164.0717 [M − H]^+^ (calculated 164.0712). IR (KBr) cm^−1^: ν_max_ 2914, 1572, 1528, 1405. UV λ_max_ (methanol) (log ε): 210 nm. ^1^H-NMR (500 MHz) (DMSO-*d*_6_): 7.43 (1H, *s*, H-4), 7.39 (2H, *d*, H-3,5), 7.30 (2H, *d*, H-2,6), 3.96 (1H, *m*, H-8), 3.22 (2H, *m*, H-7); ^13^C-NMR (125 MHz) (DMSO-*d*_6_): 173.9 (C-9), 135.2 (C-1), 129.4 (C-3,5), 129.1 (C-2, 6), 127.7 (C-4), 56.1 (C8), 36.4 (C-7).

*Latifoliusine A* (**2**). Yellowish oil; [α]_D_ = +36.1° (*c* = 0.70, methanol). HRESIMS: C_13_H_20_O_3_, *m*/*z* 247.1313 [M + Na]^+^ (calculated 247.1310). IR (KBr) cm^−1^: ν_max_ 3424, 2928, 1670, 1458, 1255. UV λ_max_ (methanol) (log ε): 232.3 nm. ^1^H- and ^13^C-NMR (500 MHz) (DMSO-*d*_6_): see Table 1.

*Dihydroxymethyl-bis(3*,*5-dimethoxy-4-hydroxyphenyl)tetrahydrofuran-9-O-β-d-glucopyranoside* (**3**). White amorphous powder; [α]_D_ = −25.4° (*c* = 1.0, methanol). HRESIMS: C_28_H_38_O_14_, *m*/*z* 597.2180 [M − H]^+^ (calculated 597.2183). IR (KBr) cm^−1^: ν_max_ 3371, 2923, 1583, 1454. UV λ_max_ (methanol) (log ε): 241, 271 nm. ^1^H-NMR (500 MHz) (DMSO-*d*_6_): 6.66 (2H, *s*, H-2,6 or 2′,6′), 6.66 (2H, *s*, H-2,6 or 2′,6′), 4.91 (1H, *d*, H-7′), 4.85 (1H, *d*, H-7), 4.16 (1H, *d*, H-1′′), 3.89, 3.56 (2H, *m*, H-9), 3.76 (12H, *s*, OCH_3_), 3.66, 3.44 (2H, *m*, H-6′′), 3.53, 3.48 (2H, *m*, H-9′), 3.18 (1H, *m*, H-5′′), 3.06 (1H, *m*, H-3′′), 3.03 (1H, *m*, H-4′′), 2.99 (1H, *m*, H-2′′), 2.12 (1H, *m*, H-8′), 2.32 (1H, *m*, H-8); ^13^C-NMR (125 MHz) (DMSO-*d*_6_): 148.4 (C-3,5 or 3′,5′), 135.1 (C-4 or 4′), 133.5 (C-1′), 133.3 (C-1), 104.3 (C-2,6 or 2′,6′), 103.6 (C-1′′), 82.6 (C-7′), 82.4 (C-7), 77.3 (C-5′′), 77.2 (C-3′′), 74.1 (C-2′′), 70.6 (C-4′′), 69.5 (C-9), 61.5 (C-6′′), 60.4(C-9′), 56.5 (OCH_3_), 53.7 (C-8), 50.7 (C-8′).

*Rel-(7R,8S,7′S,8′R)-4,9,4′,9′-tetrahydroxy-3,3′-dimethoxy-7,7′-epoxylignan 9-O-β-d-glucopyranoside* (**4**). White amorphous powder; [α]_D_ = −23.9° (*c* = 1.0, methanol). HRESIMS: C_26_H_34_O_12_, *m*/*z* 537.1969 [M − H]^+^ (calculated 537.1972). IR (KBr) cm^−1^: ν_max_ 3365, 2937, 1584, 1451. UV λ_max_ (methanol) (log ε): 233, 279 nm. ^1^H-NMR (500 MHz) (DMSO-*d*_6_): 6.94 (1H, *s*, H-2), 6.93 (1H, *s*, H-2′), 6.75 (1H, *d*, H-6), 6.74 (1H, *d*, H-6′), 6.69 (1H, *d*, H-5), 6.68 (1H, *d*, H-5′), 4.87 (1H, *d*, H-7′), 4.86 (1H, *d*, H-7), 4.16 (1H, *d*, H-1′′), 3.86, 3.53 (2H, *m*, H-9), 3.76 (6H, *s*, OCH_3_), 3.66, 3.44 (2H, *m*, H-6′′), 3.53, 3.45 (2H, *m*, H-9′), 3.12 (1H, *m*, H-5′′), 3.07 (1H, *m*, H-3′′), 3.05 (1H, *m*, H-4′′), 2.97 (1H, *m*, H-2′′), 2.31 (1H, *m*, H-8), 2.15 (1H, *m*, H-8′); ^13^C-NMR (125 MHz) (DMSO-*d*_6_): 147.8 (C-3), 147.7 (C-3′), 146.2 (C-4), 146.1 (C-4′), 134.5 (C-1), 134.2 (C-1′), 119.3 (C-6), 119.0 (C-6′), 115.5 (C-5), 115.4 (C-5′), 111.1 (C-2), 110.9 (C-2′), 103.6 (C-1′′), 82.3 (C-7′), 82.2 (C-7), 77.3 (C-5′′), 77.2 (C-3′′), 74.0 (C-2′′), 70.5 (C-4′′), 69.3 (C-9), 61.5 (C-6′′), 60.4 (C-9′), 56.1 (OCH_3_), 53.6 (C-8), 50.7 (C-8′).

*Apigenin 6-C-α-l-arabinopyranosyl-8-C-β-d-glucopyranoside* (**5**). Yellow amorphous powder; HRESIMS: C_26_H_28_O_14_, *m*/*z* 563.1400 [M − H]^+^ (calculated 563.1401). IR (KBr) cm^−1^: ν_max_ 3386, 2954, 1706, 1573, 1467. UV λ_max_ (methanol) (log ε): 271, 334 nm. ^1^H-NMR (500 MHz) (DMSO-*d*_6_): 7.95 (2H, *d*, H-2′,6′), 6.93 (2H, *d*, H-3′,5′), 6.81 (1H, *s*, H-3), 4.81 (1H, *d*, H-1′′′), 4.62 (1H, *d*, H-1′′), 4.00 (1H, *m*, H-2′′), 3.89 (1H, *m*, H-2′′′), 3.79, 3.57 (2H, *m*, H-5′′), 3.77 (1H, *m*, H-4′′), 3.74, 3.52 (2H, *m*, H-6′′′), 3.42 (1H, *m*, H-3′′), 3.36 (1H, *m*, H-4′′′), 3.30 (1H, *m*, H-3′′′), 3.27 (1H, *m*, H-5′′′); ^13^C-NMR (125 MHz) (DMSO-*d*_6_): 180.8 (C-4), 162.8 (C-7), 161.5 (C-2), 160.6 (C-4′), 159.4 (C-5), 154.5 (C-9), 128.7 (C-2′,6′), 121.9 (C-1′), 115.7 (C-3′,5′), 110.1 (C-6), 104.5 (C-8), 102.0 (C-3), 100.1 (C-10), 81.5 (C-5′′′), 78.8 (C-3′′′), 74.1 (C-1′′), 74.0 (C-3′′), 73.8 (C-1′′′), 71.0 (C-2′′′), 70.4 (C-4′′′), 69.7 (C-5′′), 69.0 (C-2′′), 68.5 (C-4′′), 61.0 (C-6′′′).

*Apigenin 7-O,8-C-di-glucopyranoside* (**6**). Yellow amorphous powder; HRESIMS: C_26_H_34_O_12_, *m*/*z* 563.1405 [M − H]^+^ (calculated 563.1401). IR (KBr) cm^−1^: ν_max_ 3331, 2835, 1725, 1544, 1486. UV λ_max_ (methanol) (log ε): 271, 336 nm. ^1^H-NMR (500 MHz) (DMSO-*d*_6_): 7.93 (2H, *d*, H-2′,6′), 6.94 (2H, *d*, H-3′,5′), 6.79 (1H, *s*, H-3), 6.19 (1H, *s*, H-8), 4.88 (1H, *d*, H-1′′′), 4.64 (1H, *d*, H-1′′), 4.02 (1H, *m*, H-2′′), 3.79 (1H, *m*, H-4′′), 3.77, 3.55 (2H, *m*, H-5′′), 3.60, 3.44 (2H, *m*, H-6′′′), 3.43 (1H, *m*, H-3′′), 3.23 (1H, *m*, H-3′′′), 3.22 (1H, *m*, H-2′′′), 3.20 (1H, *m*, H-4′′′), 3.05 (1H, *m*, H-5′′′); ^13^C-NMR (125 MHz) (DMSO-*d*_6_): 181.0 (C-4), 164.6 (C-7), 162.2 (C-2), 160.7 (C-4′), 159.6 (C-5), 155.0 (C-9), 128.6 (C-2′,6′), 122.0 (C-1′), 115.9 (C-3′,5′), 110.4 (C-6), 105.3 (C-10), 103.4 (C-1′′′), 102.3 (C-3), 92.5 (C-8), 77.6 (C-5′′′), 77.0 (C-3′′′), 74.3 (C-1′′), 74.2 (C-3′′), 72.5 (C-2′′′), 70.4 (C-4′′′), 69.8 (C-5′′), 69.0 (C-4′′), 68.5 (C-2′′), 61.4 (C-6′′′).

*(7S,8R) Syringylglycerol-8-O-4′-sinapyl ether 4-O-β-d-glucopyranoside* (**7**). Yellow amorphous powder; [α]_D_ = −15.9° (*c* = 1.0, methanol). HRESIMS: C_28_H_38_O_14_, *m*/*z* 597.2179 [M − H]^−^ (calculated 597.2183). IR (KBr) cm^−1^: *ν*_max_ 3381, 2927, 1654, 1451, 1253. UV λ_max_ (methanol) (log ε): 230 nm, 270 nm. CD (*c* 1.0 × 10^−3^, MeOH): ∆ε_205nm_ +9.4440, ∆ε_243.5nm_ −0.4727, ∆ε_282.0nm_ +0.1389. ^1^H- and ^13^C-NMR (500 MHz) (DMSO-*d*_6_): see Table 1.

*(7S,8S) Syringylglycerol-8-O-4′-sinapyl ether 7-O-β-d-glucopyranoside* (**8**). Yellow amorphous powder; [α]_D_ = −3.11° (*c* = 1.0, methanol). HRESIMS: C_28_H_38_O_14_, *m*/*z* 633.1952 [M + Cl]^−^ (calculated 633.1950). IR (KBr) cm^−1^: ν_max_ 3385, 2927, 1584, 1460. UV λ_max_ (methanol) (log ε): 230 nm, 269 nm. CD (*c* 1.0 × 10^−3^, MeOH): ∆ε_212.5nm_ +6.6316, ∆ε_237.5nm_ +2.0655, ∆ε_285.0nm_ −0.5970. ^1^H- and ^13^C-NMR (500 MHz) (DMSO-*d*_6_): see Table 1.

*(7S,8S) Syringylglycerol-8-O-4′-sinapyl ether 9′-O-β-d-glucopyranoside* (**9**). White amorphous powder; [α]_D_ = −9.5° (*c* = 1.0, methanol). HRESIMS: C_28_H_38_O_14_, *m*/*z* 597.2181 [M – H]^+^ (calculated 597.2183). IR (KBr) cm^−1^: ν_max_ 3379, 2931, 1582, 1464. UV λ_max_ (methanol) (log ε): 230, 270 nm. ^1^H-NMR (500 MHz) (DMSO-*d*_6_): 6.75 (2H, *s*, H-2′,6′), 6.60 (1H, *s*, H-2,6), 6.57 (1H, *d*, H-7′), 6.34 (1H, *dt*, H-8′), 4.81 (1H, *dd*, H-7), 4.41, 4.19 (1H, *d*, H-9′), 4.15 (1H, *m*, H-8), 4.21 (1H, *d*, H-1′′), 3.68, 3.40 (1H, *m*, H-9), 3.67, 3.44 (2H, *m*, H-6′′), 3.14 (1H, *m*, H-5′′), 3.09 (1H, *m*, H-3′′), 3.07 (1H, *m*, H-4′′), 3.05 (1H, *m*, H-2′′); ^13^C-NMR (125 MHz) (DMSO-*d*_6_): 152.8 (C-3′′,5′′), 147.6 (C-3,5), 135.5 (C-4′), 134.4 (C-4), 132.6 (C-1), 132.0 (C-1′), 131.4 (C-7′), 125.8 (C-8′), 104.4 (C-2,6), 103.9 (C-2′,6′), 102.2 (C-1′′), 86.4 (C-8), 77.0 (C-3′′), 76.9 (C-5′′), 73.6 (C-2′′), 72.4 (C-7), 70.2 (C-4′′), 68.7 (C-9′), 61.2 (C-6′′), 59.9 (C-9).

*(7R,8S) Syringylglycerol-8-O-4′-sinapyl ether 7-O-β-d-glucopyranoside* (**10**). Yellow amorphous powder; [α]_D_ = +4.55° (*c* = 1.0, methanol). HRESIMS: C_28_H_38_O_14_, *m*/*z* 633.1953 [M + Cl]^−^ (calculated 633.1950). IR (KBr) cm^−1^: ν_max_ 3387, 2929, 1585, 1461. UV λ_max_ (methanol) (log ε): 230 nm, 271 nm. CD (*c* 1.0 × 10^−3^, MeOH): ∆ε _207.5 nm_ +4.6915, ∆ε _231.0 nm_ +1.6611, ∆ε _272.0 nm_ ‒1.7423. ^1^H- and ^13^C-NMR (500 MHz) (DMSO-*d*_6_): see Table 1.

### 3.6. Antibacterial Activity Assay

#### 3.6.1. Microbial Strains

Two food contaminating bacteria *Staphylococcus aureus* (Gram (+)) and *Escherichia coli* (Gram (−)) were selected as test strains, another Gram -ositive bacteria (*Bacillus thuringiensis*) along with another Gram-negative bacteria (*Pseudomonas solanacearum*) were selected for testing the antibacterial selectiveness of isolated compounds, all four bacteria strains were obtained from the Agricultural Product Key Laboratory of Anhui Agriculture University, Hefei City, Anhui, China.

#### 3.6.2. Antibacterial Screening

The concentrations of the compounds used for the antibacterial screening experiments were 6.2 mg/mL (**1**), 7.0 mg/mL (**2**), 30.0 mg/mL (**3**), 21.0 mg/mL (**4**), 20.0 mg/mL (**5**), 13.6 mg/mL (**6**), 6.92 mg/mL (**7**), 6.88 mg/mL (**8**), 21.0 mg/mL (**9**) and 20.0 mg/mL (**10**). The concentrations were set for simulating the content ratio in *Indocalamus latifolius* McClure Leaves, which were determined in our preliminary research. Briefly, 200 μL of a suspension containing 10^8^ colony-forming units (CFU)/mL of bacteria was spread onto nutrient agar (NA). The disks (6 mm in diameter) were impregnated with 10 μL of different concentrations of the compounds (dissolved in water-ethanol) and placed on the inoculated agar. Negative controls were prepared using water and ethanol. Ampicillin sodium (5 μg/disc) was used as the positive control. The inoculated plates of bacteria were incubated at 37 °C for 24 h. The antibacterial activity was evaluated by measuring the zone of inhibition.

## 4. Conclusions

Since ancient times, the leaves of *I. latifolius* McClure have been used as a packaging material for food, and presently, they still play a unique role in producing “Zongzi” in China. The identification of the antibacterial compounds in the leaves of *I. latifolius* McClure is important for helping us to understand the long shelf life of “Zongzi” as well as for exploring the potential of *I. latifolius* McClure leaves as a natural, healthy, and eco-friendly alternative packaging material for other applications.

## Supplementary Materials

^1^H, ^13^C, HSQC, HMBC and NOESY spectra of latifoliusine A (**2**), ^1^H, ^13^C, HSQC and HMBC spectra of (7*S*,8*R*) syringylglycerol-8-*O*-4′-sinapyl ether 4-*O*-β-d-glucopyranoside (**7**) and (7*S*,8*S*) syringylglycerol-8-*O*-4′-sinapyl ether 7-*O*-β-d-glucopyranoside (**8**), ^1^H and ^13^C spectra of (7*R*,8*S*) syringylglycerol-8-*O*-4′-sinapyl ether 7-*O*-β-d-glucopyranoside (**10**) in DMSO. IR, HRESIMS and UV spectra of latifoliusine A (**2**); IR, HRESIMS, CD and UV spectra of (7*S*,8*R*) syringylglycerol-8-*O*-4′-sinapyl ether 4-*O*-β-d-glucopyranoside (**7**), (7*S*,8*S*) syringylglycerol-8-*O*-4′-sinapyl ether 7-*O*-β-d-glucopyranoside (**8**), (7*R*,8*S*) syringylglycerol-8-*O*-4′-sinapyl ether 7-*O*-β-d-glucopyranoside (**10**). Supplementary materials can be accessed at: http://www.mdpi.com/1420-3049/20/09/15686/s1.
