# Supplementary material for: Chemical Constituents and Antibacterial Properties of Indocalamus latifolius McClure Leaves, the Packaging Material for “Zongzi”
Source: Molecules. 2015 Aug 28;20(9):15686–700. doi: 10.3390/molecules200915686 (PMC6331938; doi:10.3390/molecules200915686)
Supplement: Supplementary file 1 [file molecules-20-15686-s001.pdf]

Supplementary Materials

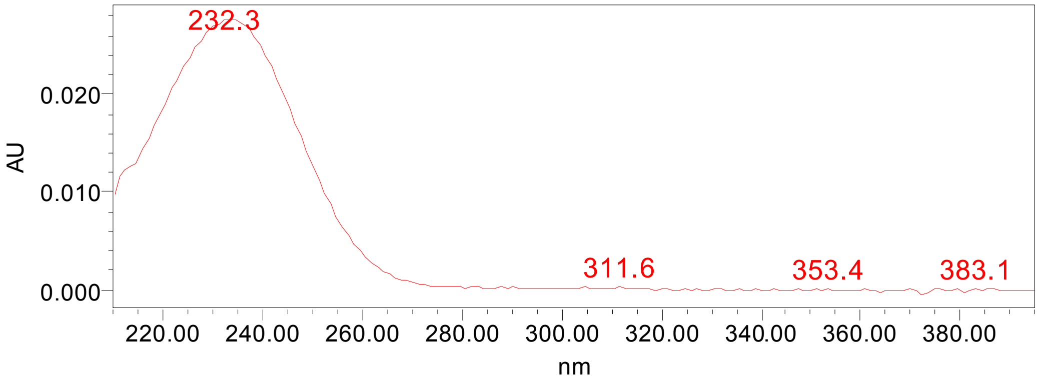

Figure S1. UV of compound **2**.

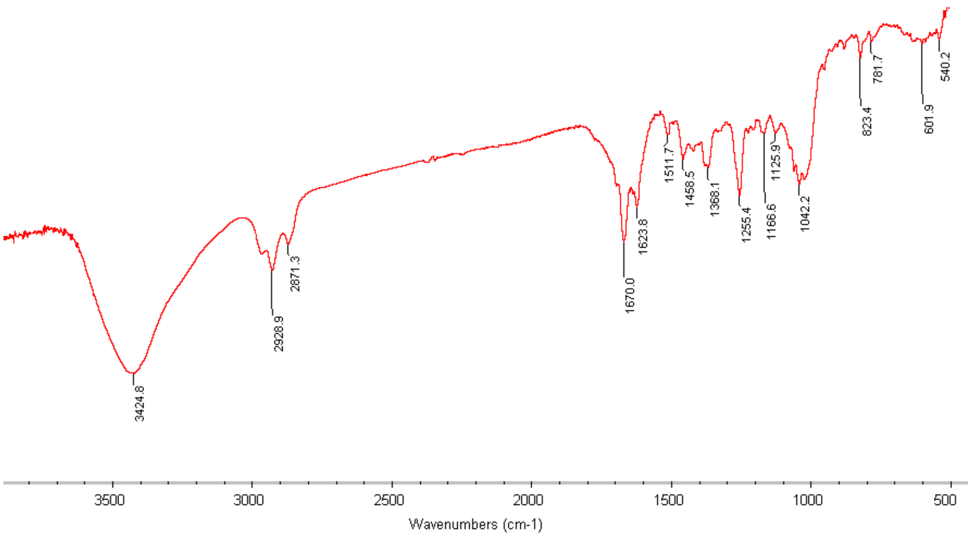

Figure S2. IR of compound **2**.

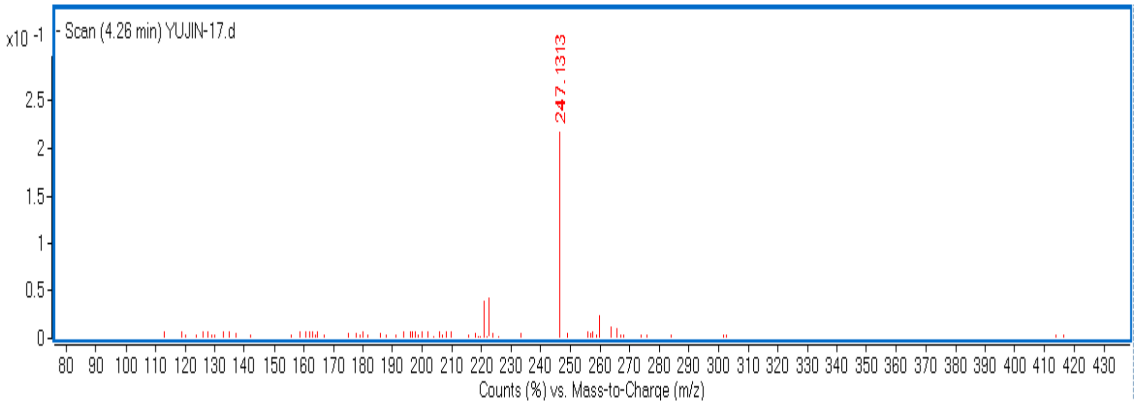

Figure S3. HRESIMS of compound **2**.

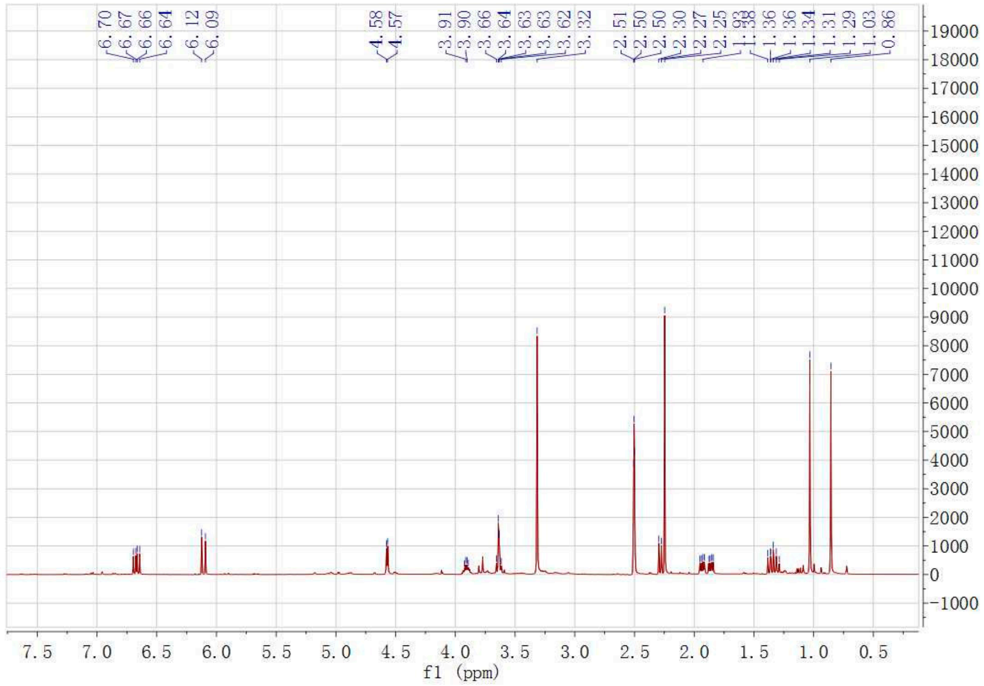

Figure S4. ^{1}H-NMR of compound **2** (Data acquired in DMSO-d_6 at 500 MHz).

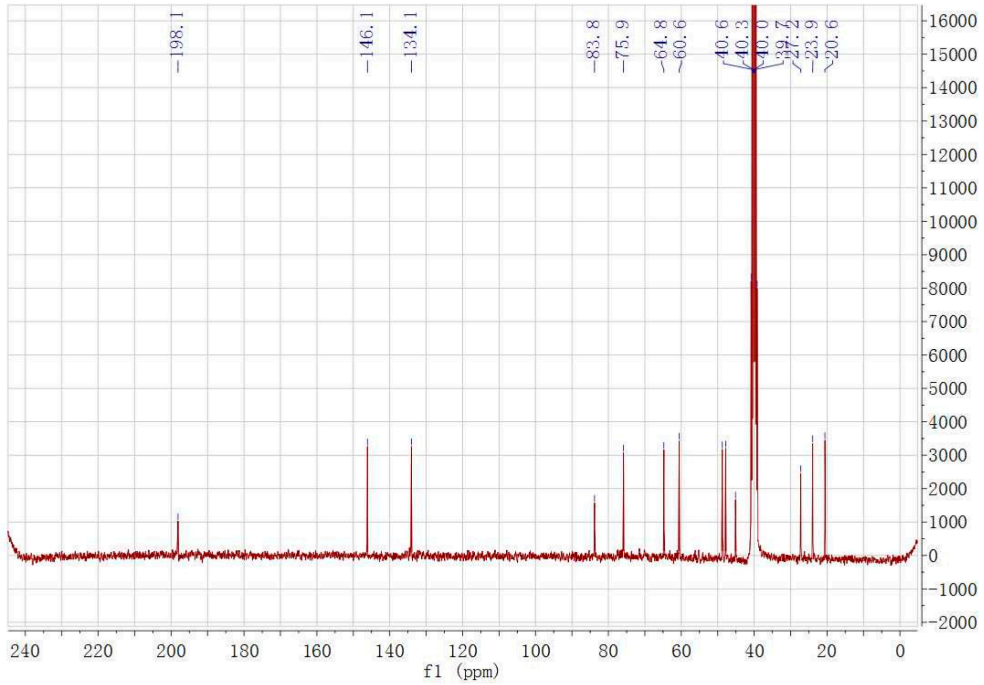

Figure S5. ^{13}C-NMR of compound **2** (Data acquired in DMSO-d_6 at 500 MHz).

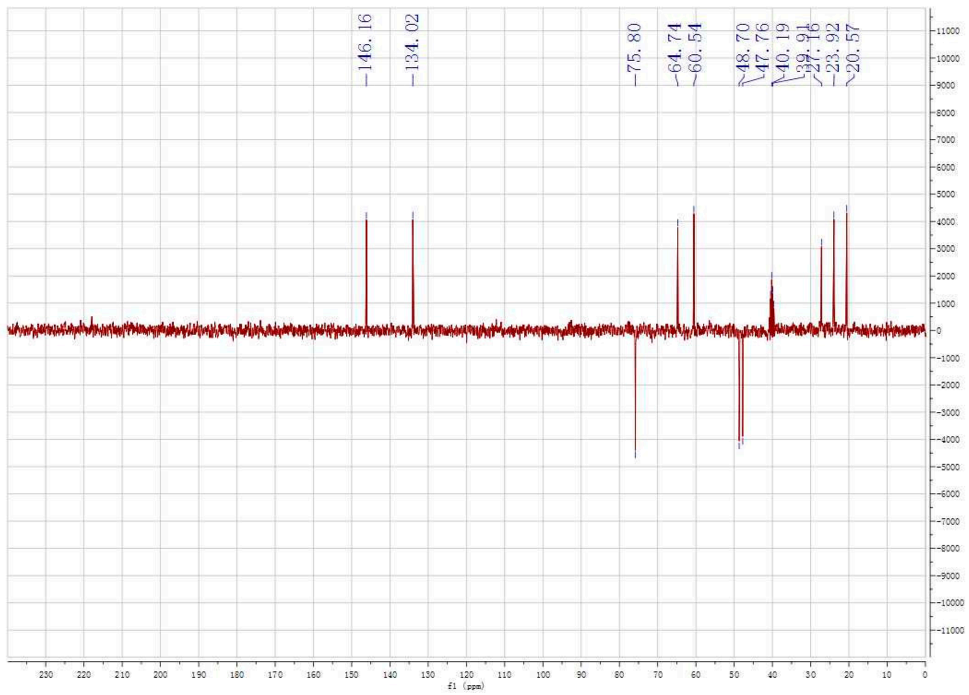

Figure S6. Dept 135° of compound **2** (Data acquired in DMSO-d_6 at 500 MHz).

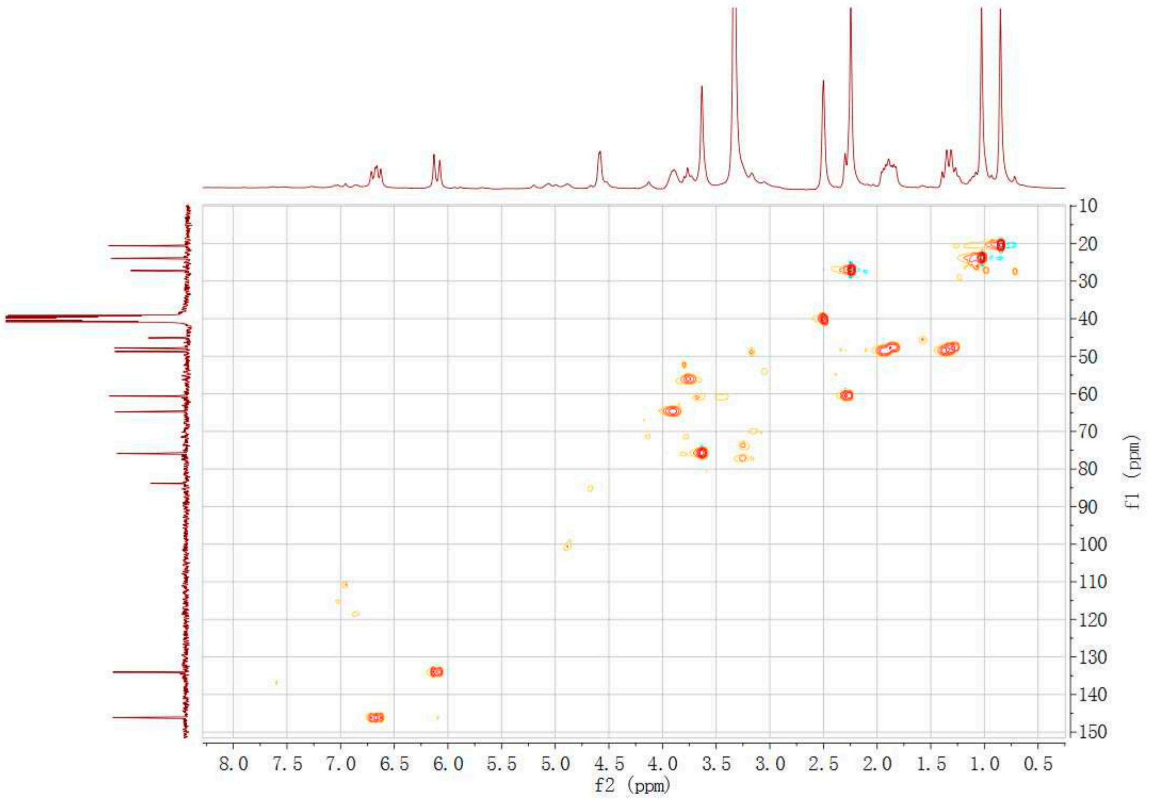

Figure S7. HSQC of compound **2** (Data acquired in DMSO-d_6 at 300 MHz).

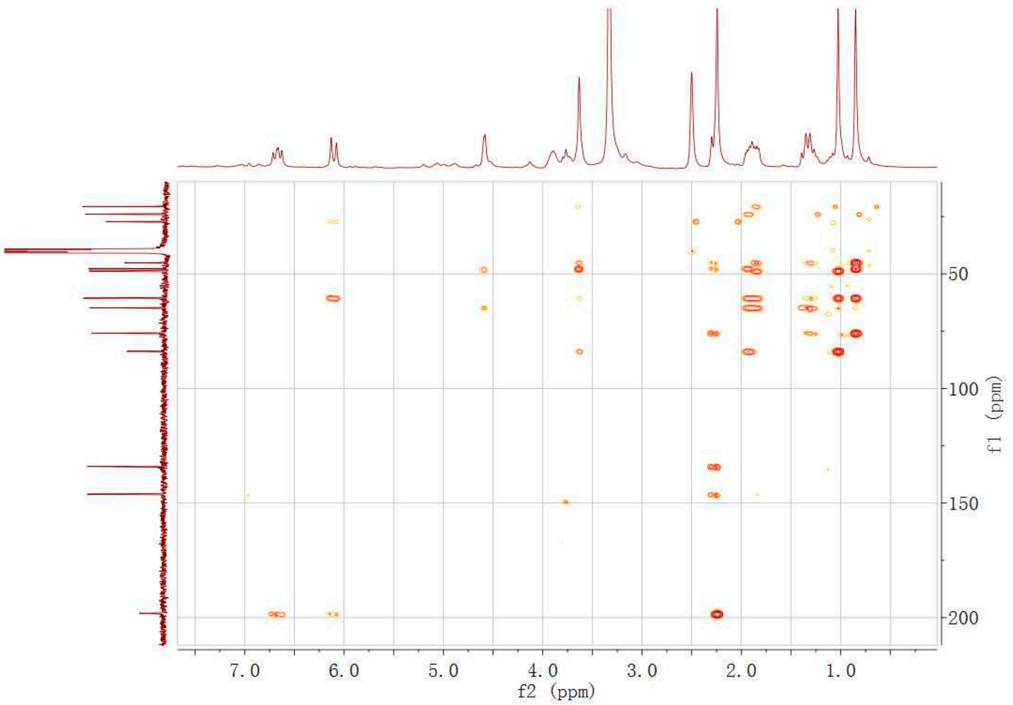

Figure S8. HMBC of compound **2** (Data acquired in DMSO-d_6 at 300 MHz).

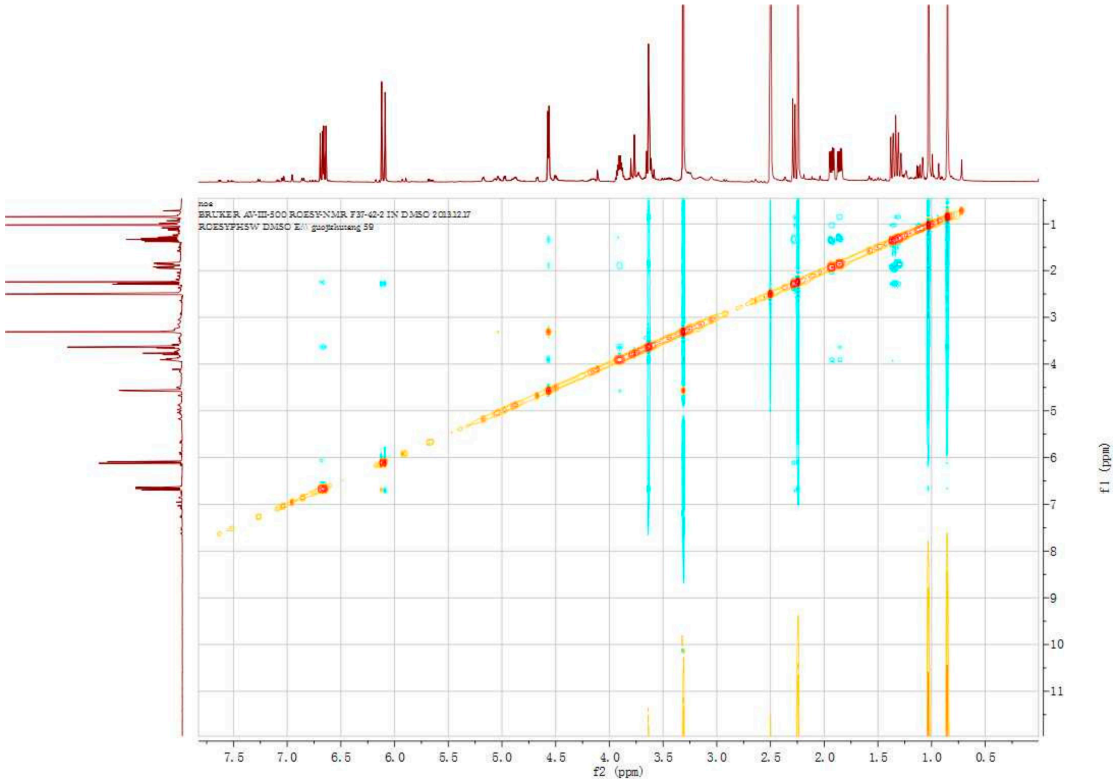

Figure S9. NOESY of compound **2** (Data acquired in DMSO-d_6 at 500 MHz).

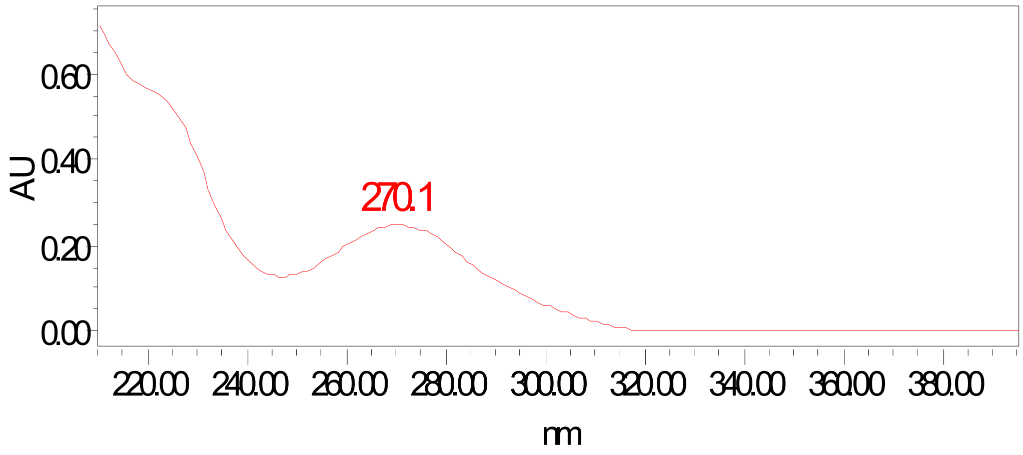

Figure S10. UV of compound **7**.

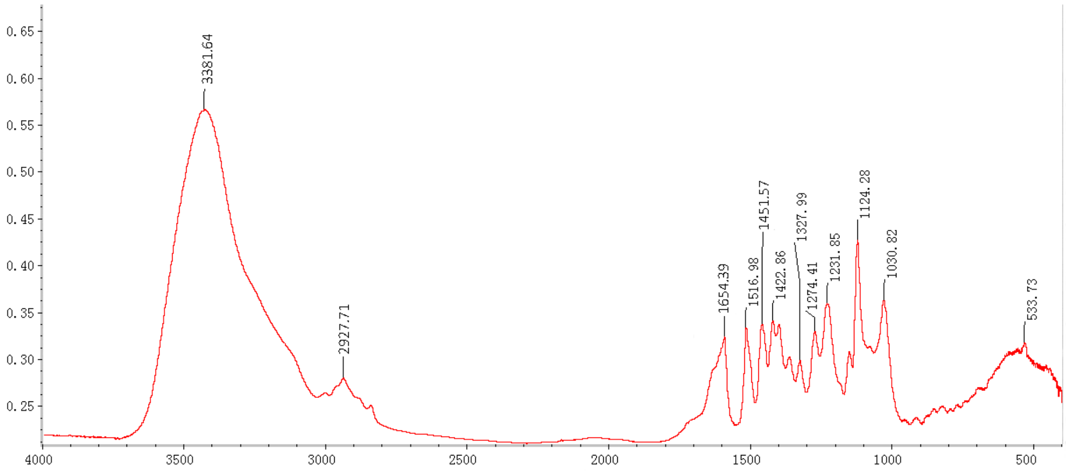

Figure S11. IR of compound **7**.

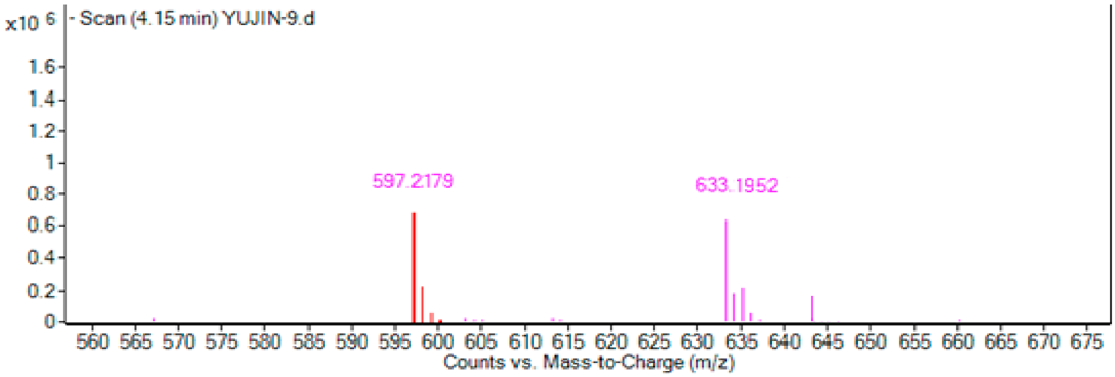

Figure S12. HRESIMS of compound **7**.

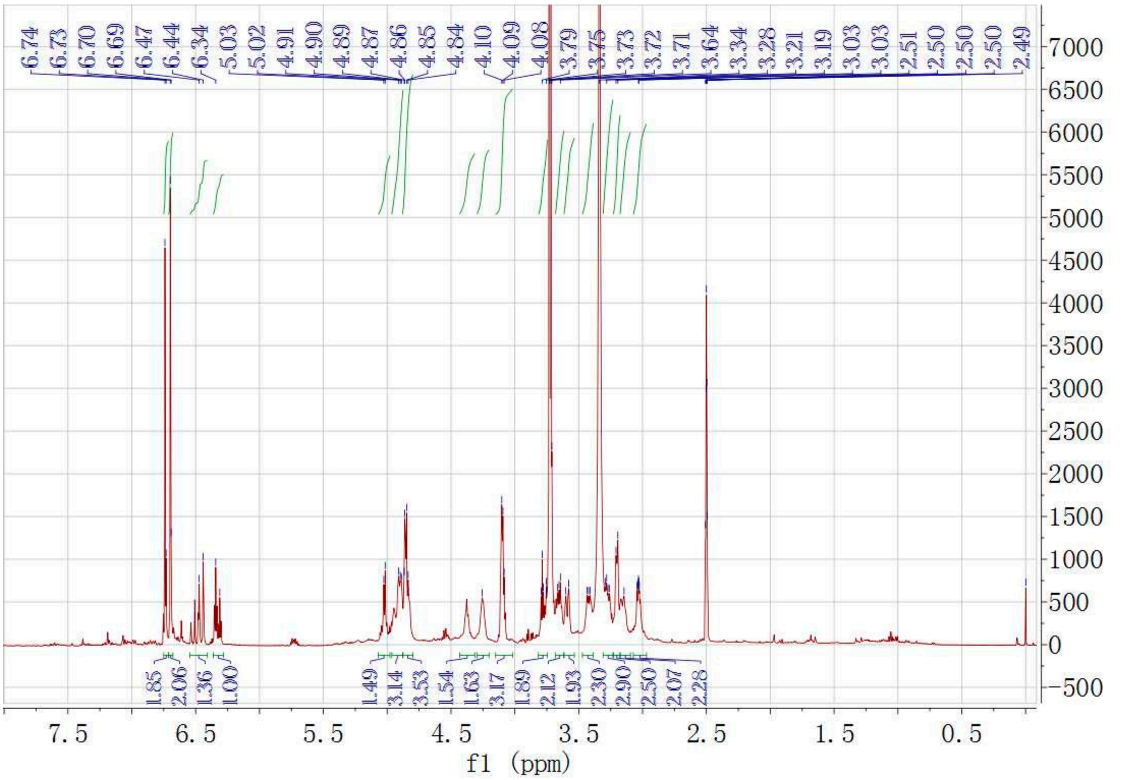

Figure S13. ^{1}H-NMR compound **7** (Data acquired in DMSO-d_6 at 500 MHz).

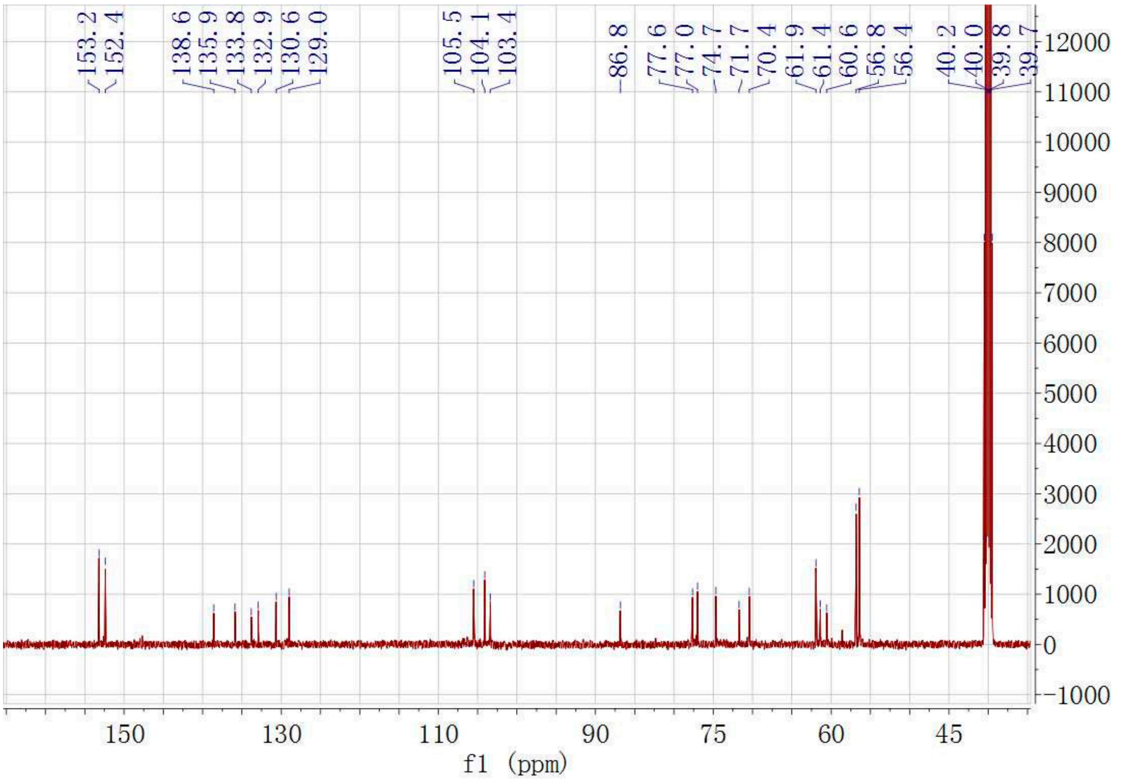

Figure S14. ^{13}C-NMR compound **7** (Data acquired in DMSO-d_6 at 500 MHz).

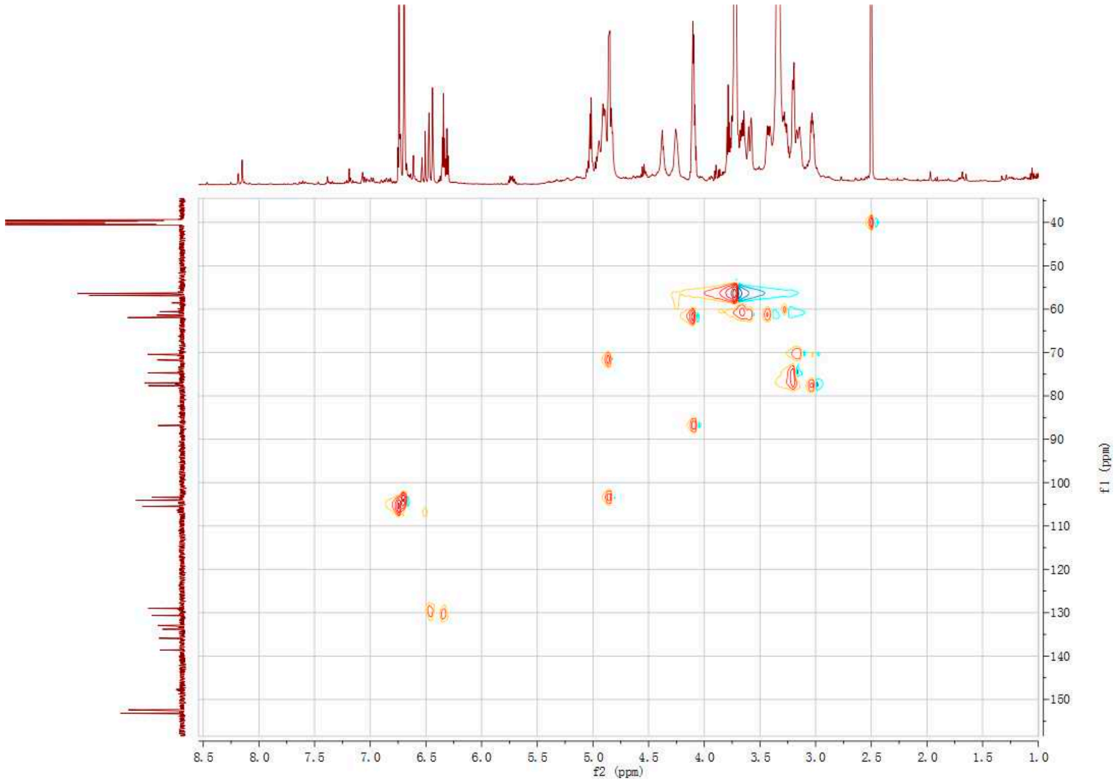

Figure S15. HSQC compound **7** (Data acquired in DMSO-d_6 at 300 MHz).

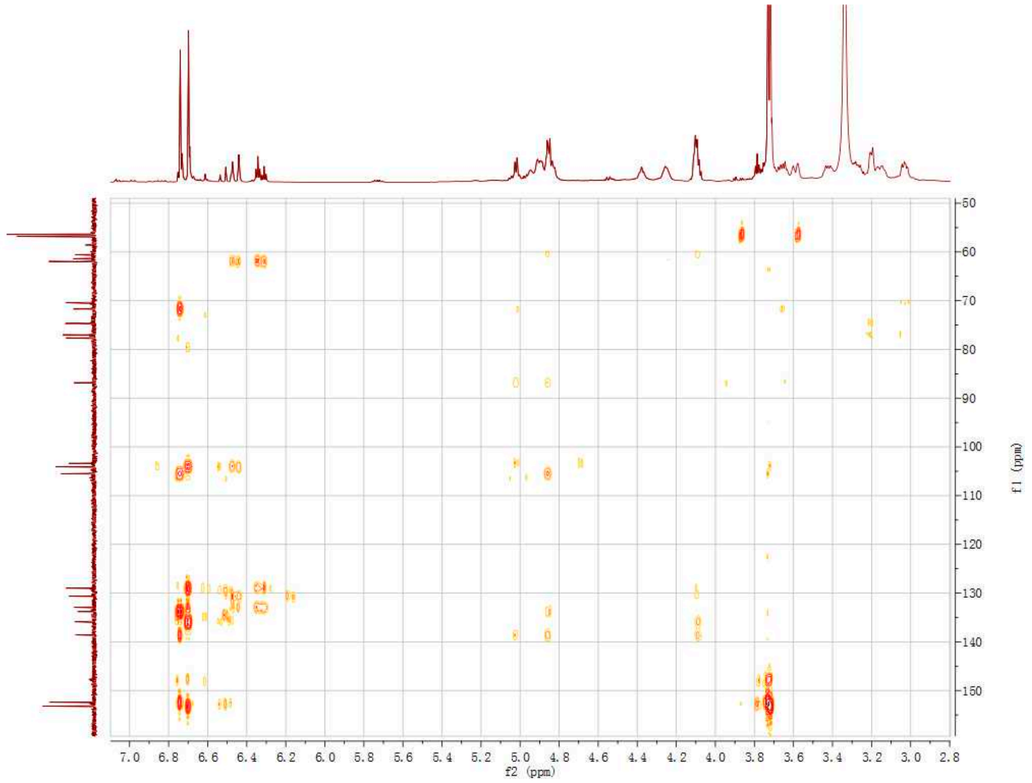

Figure S16. HMBC compound **7** (Data acquired in DMSO-d_6 at 300 MHz).

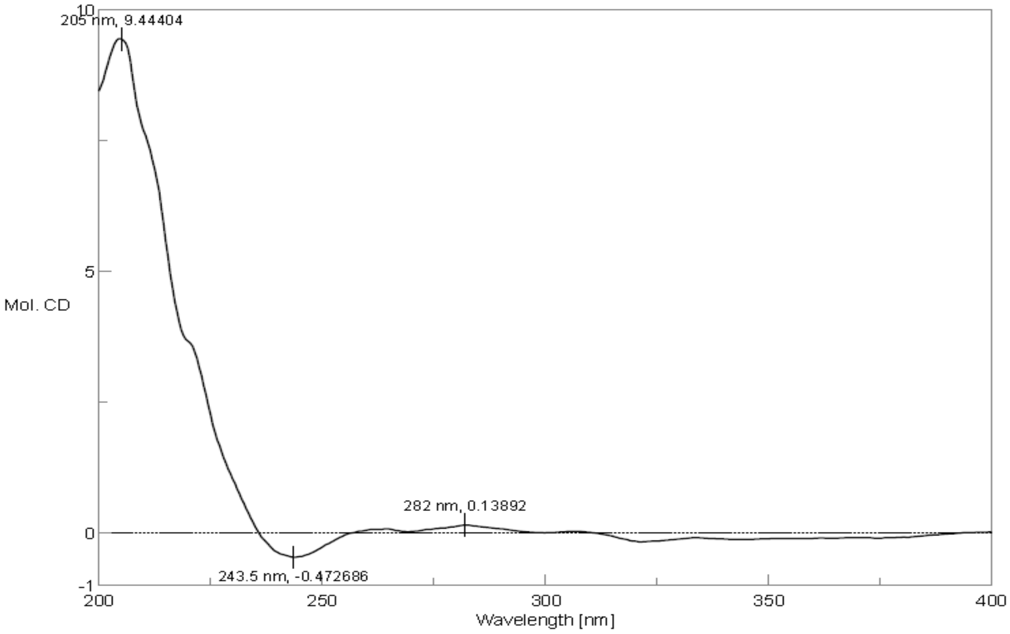

Figure S17. CD of compound **7**.

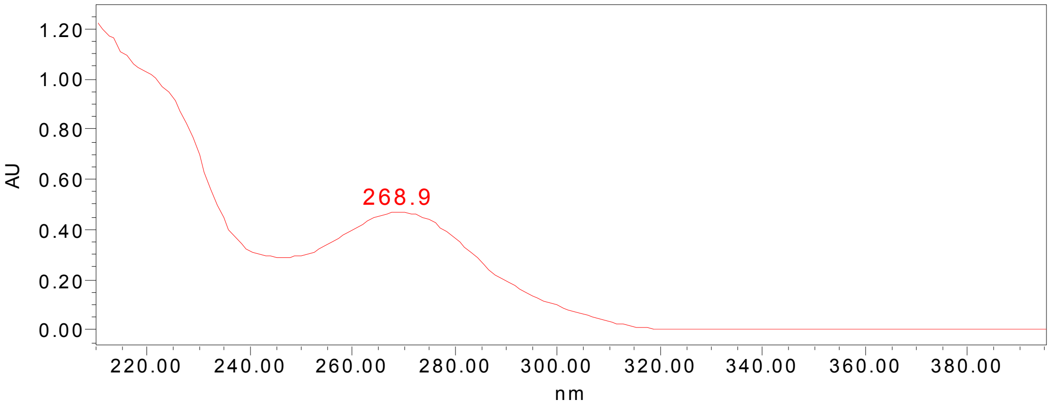

Figure S18. UV of compound **8**.

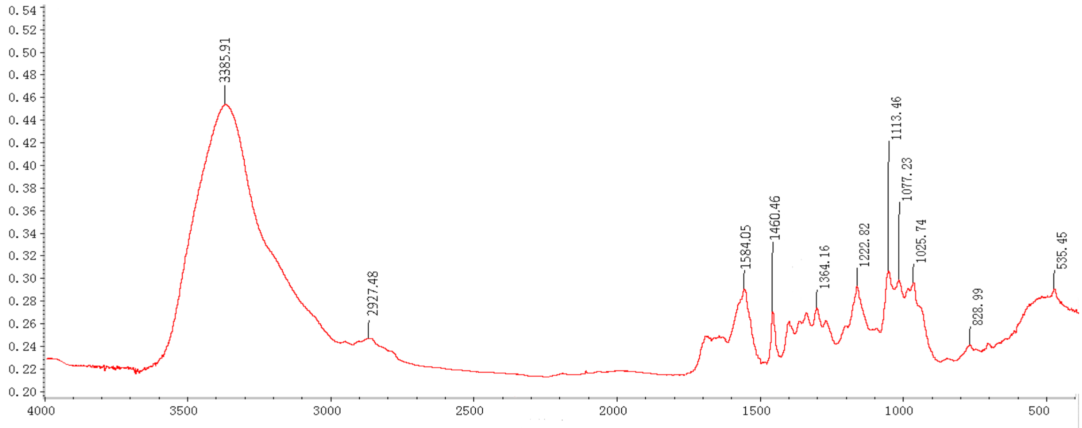

Figure S19. IR of compound **8**.

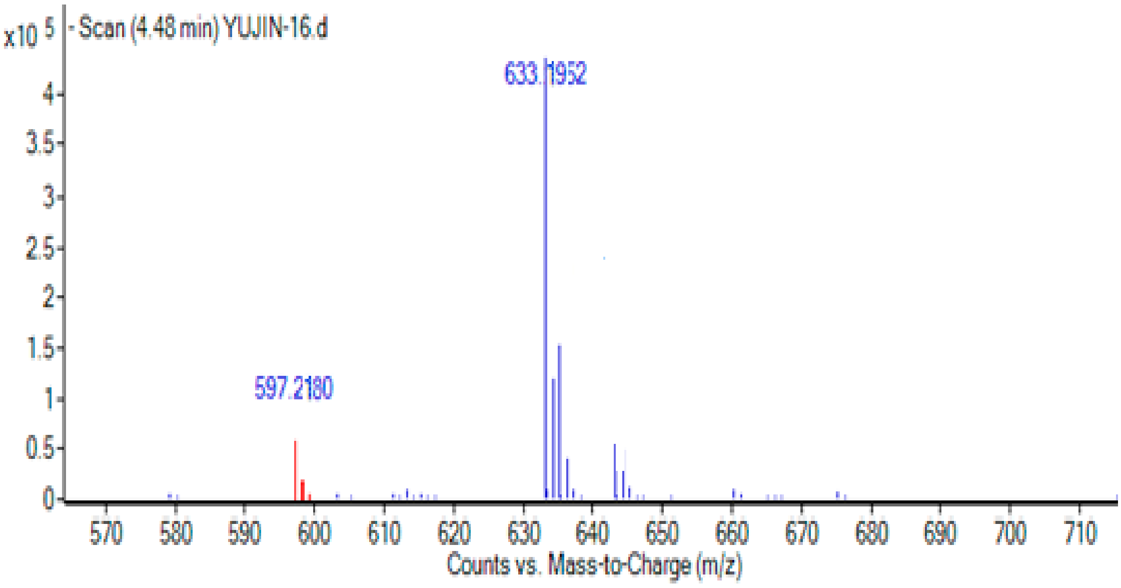

Figure S20. HRESIMS of compound **8**.

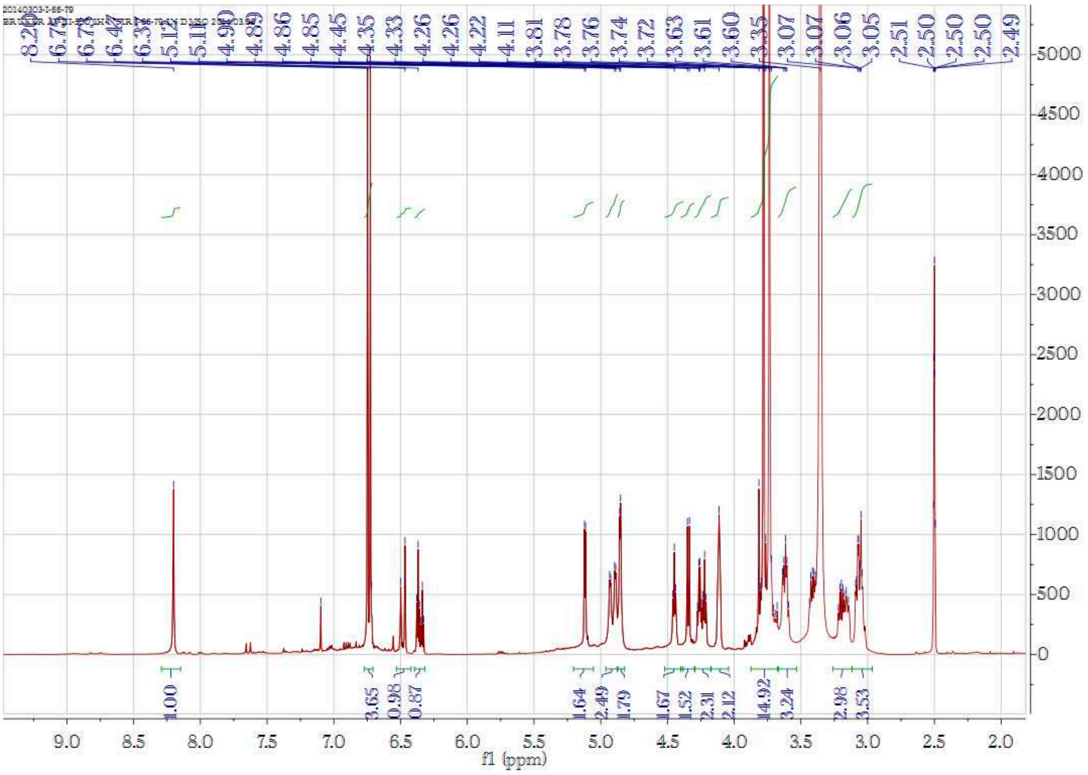

Figure S21. ^{1}H-NMR of compound **8** (Data acquired in DMSO-d_6 at 500 MHz).

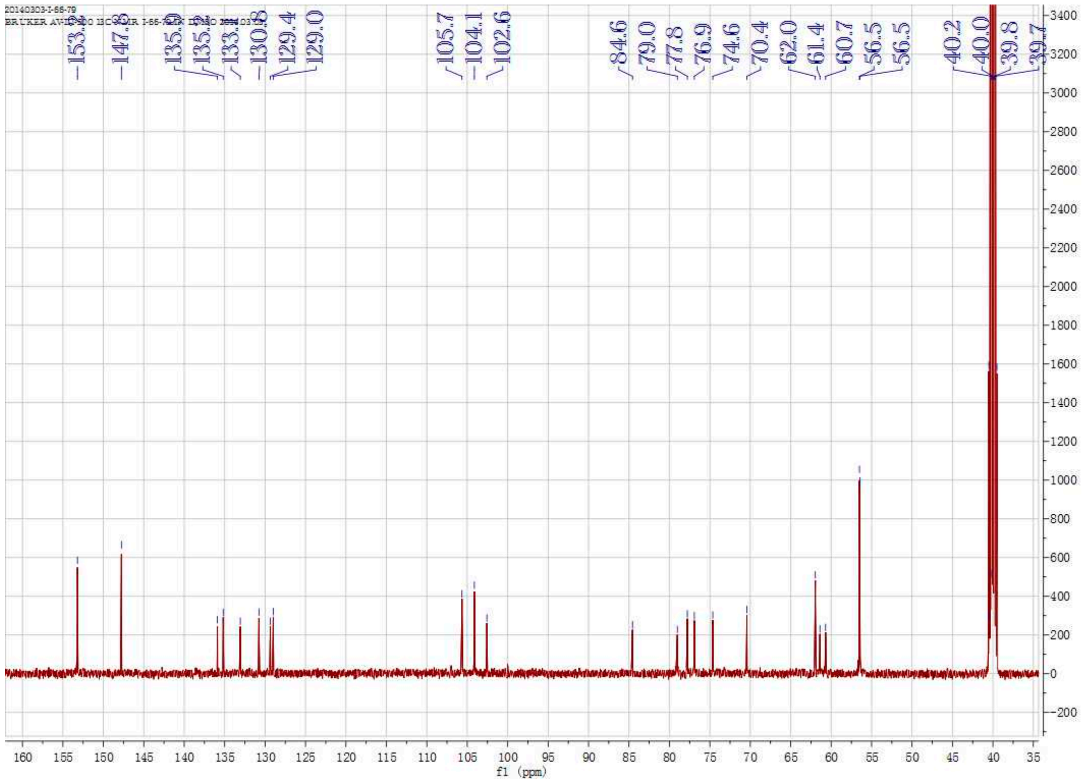

Figure S22. ^{13}C-NMR of compound **8** (Data acquired in DMSO-d_6 at 500 MHz).

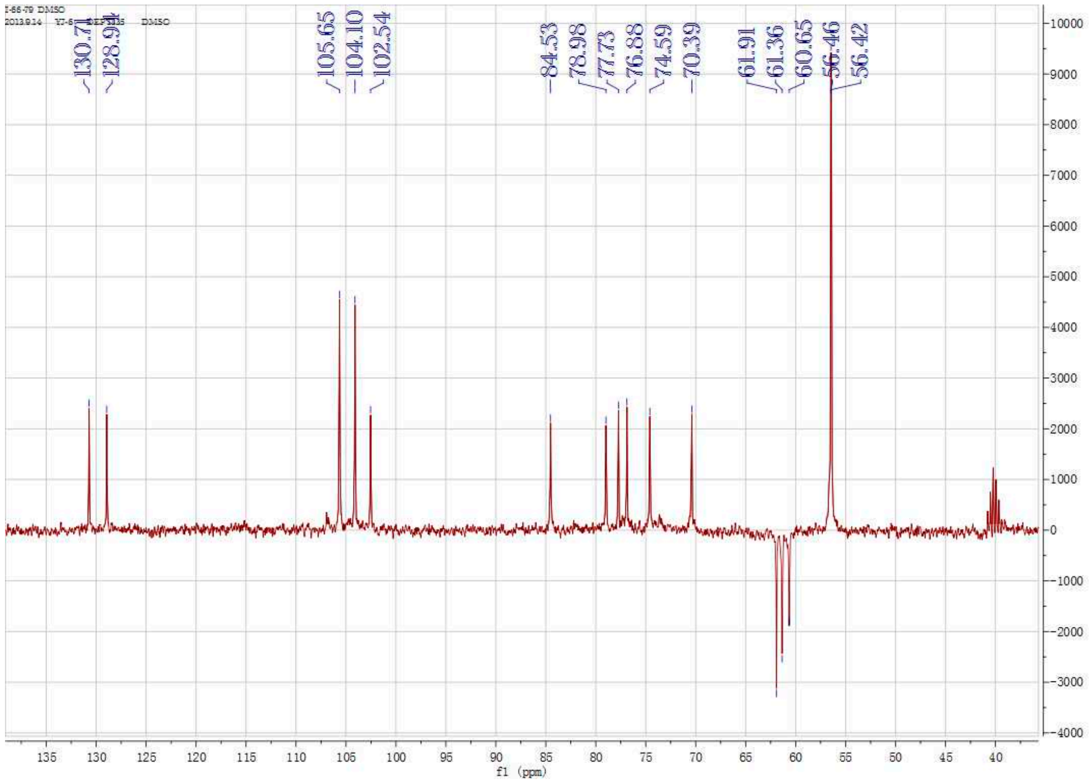

Figure S23. Dept 135° of compound **8** (Data acquired in DMSO-d_6 at 300 MHz).

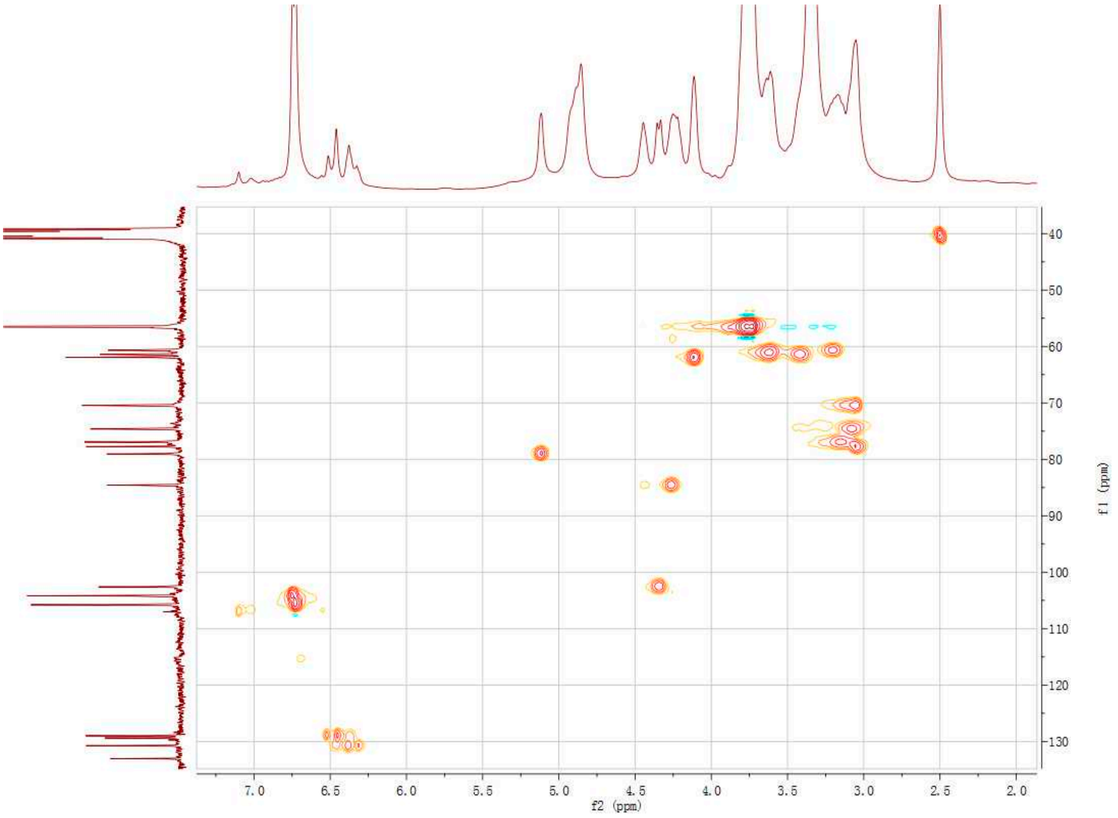

Figure S24. HSQC of compound **8** (Data acquired in DMSO-d_6 at 300 MHz).

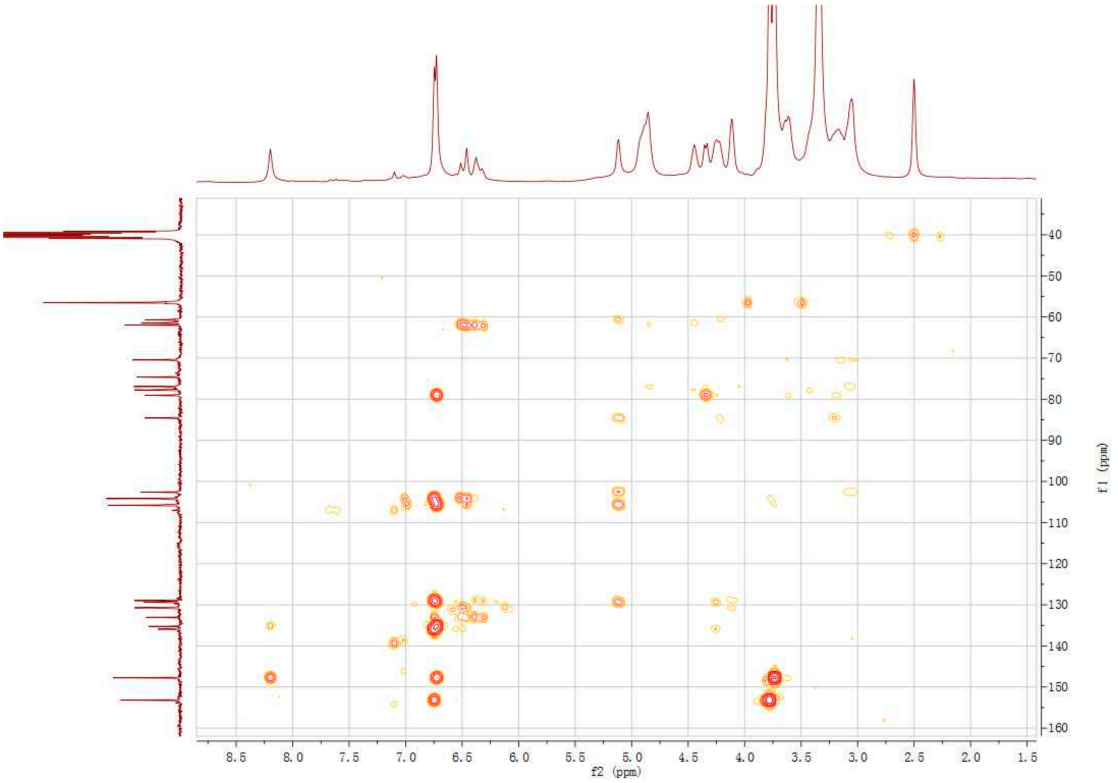

Figure S25. HMBC of compound **8** (Data acquired in DMSO-d_6 at 300 MHz).

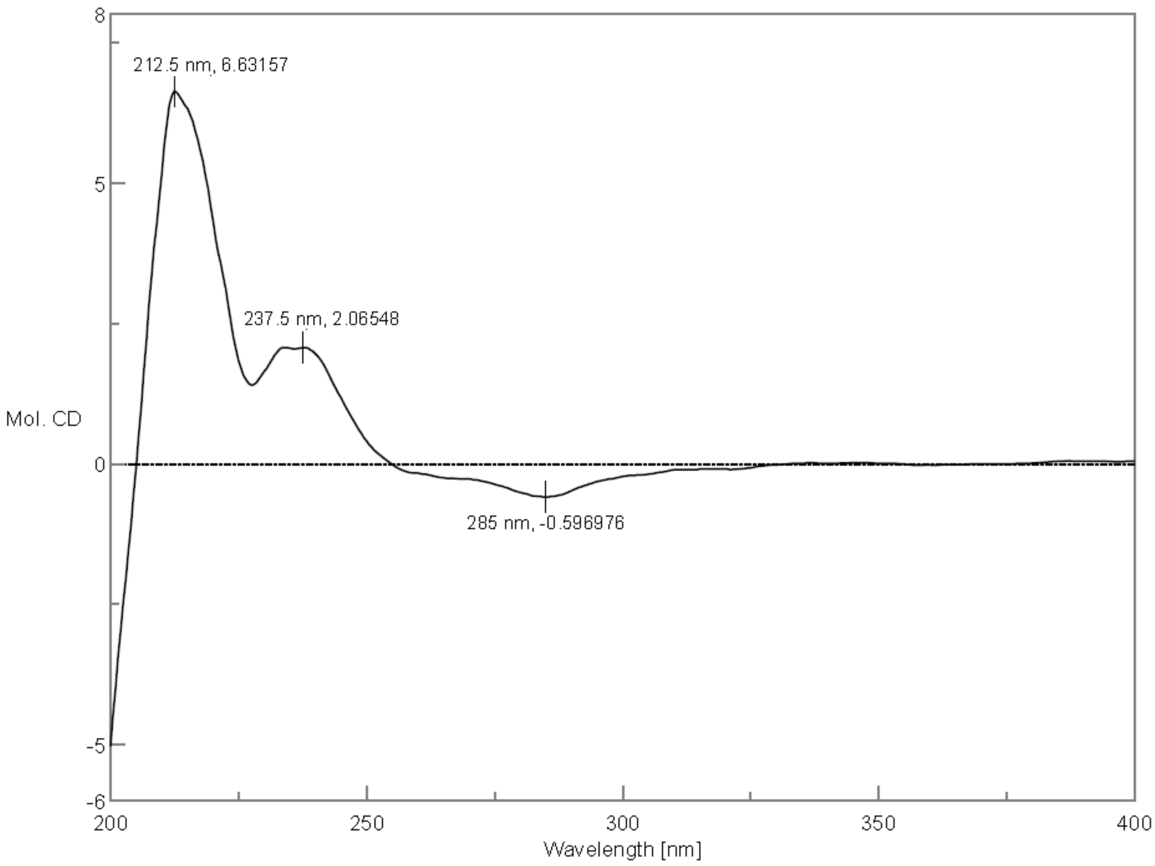

Figure S26. CD of compound **8**.

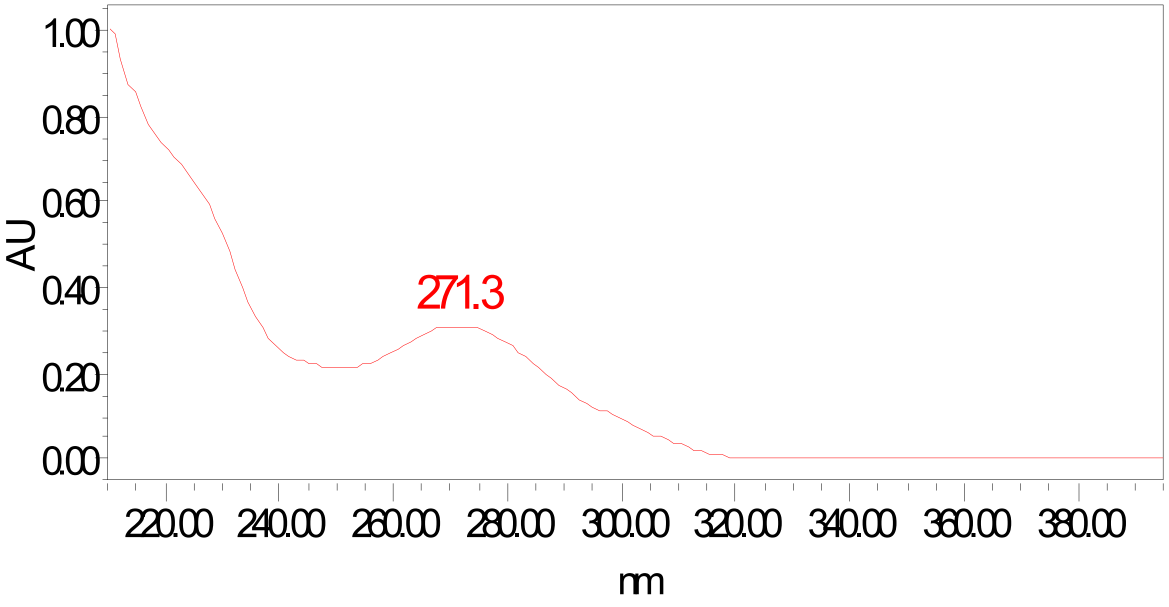

Figure S27. UV of compound **10**.

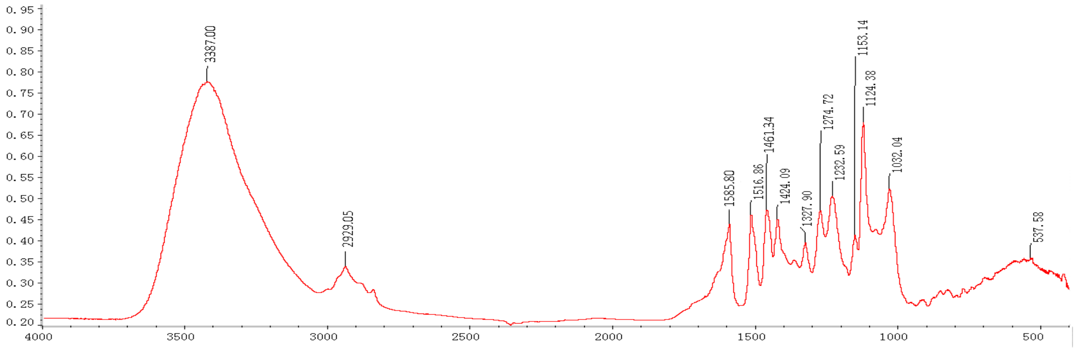

Figure S28. IR of compound **10**.

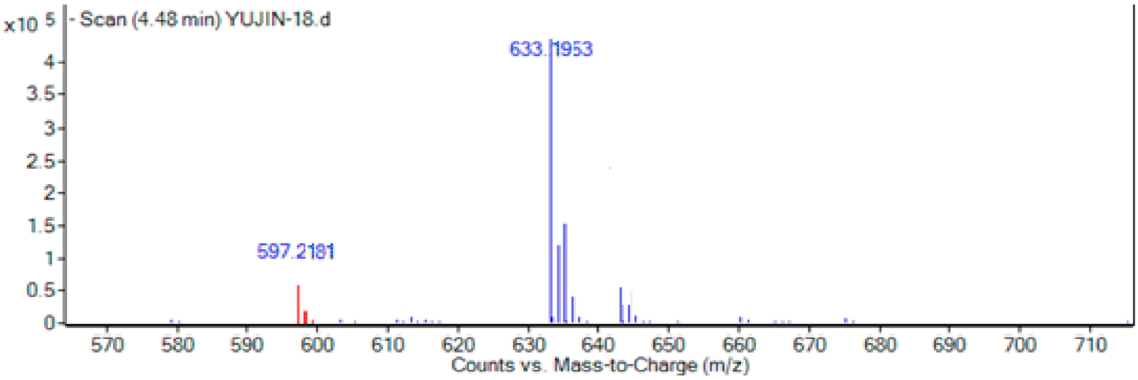

Figure S29. HRESIMS of compound **10**.

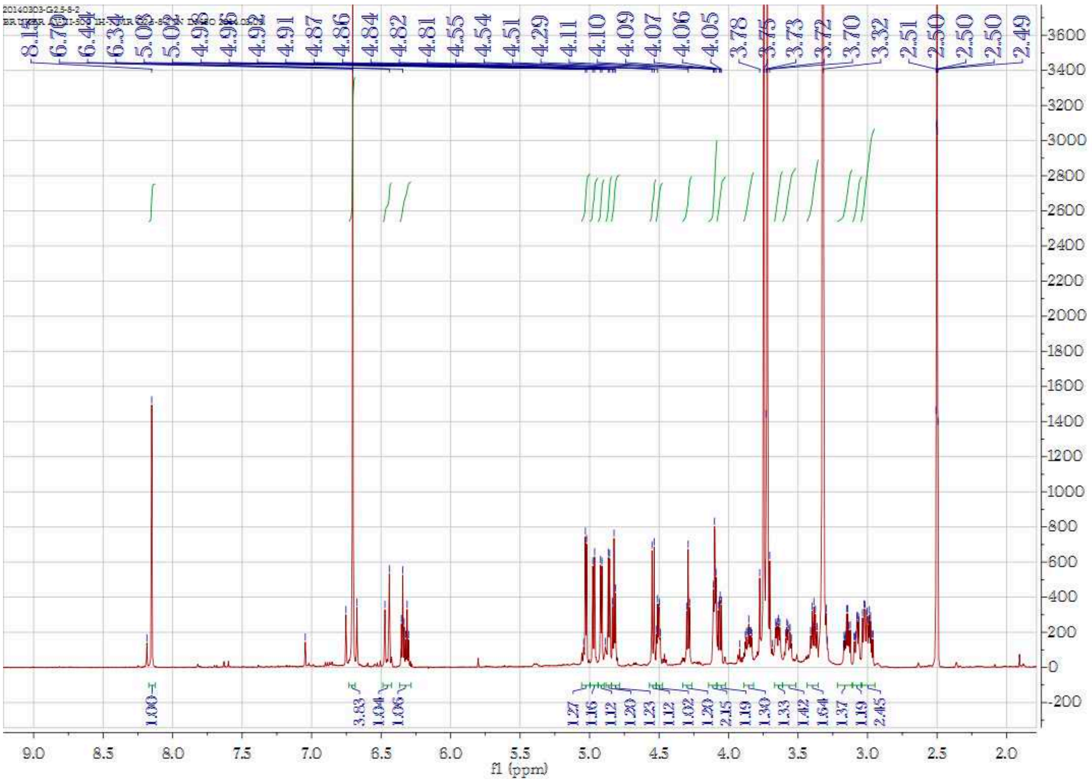

Figure S30. ^{1}H-NMR of compound **10** (Data acquired in DMSO-d_6 at 500 MHz).

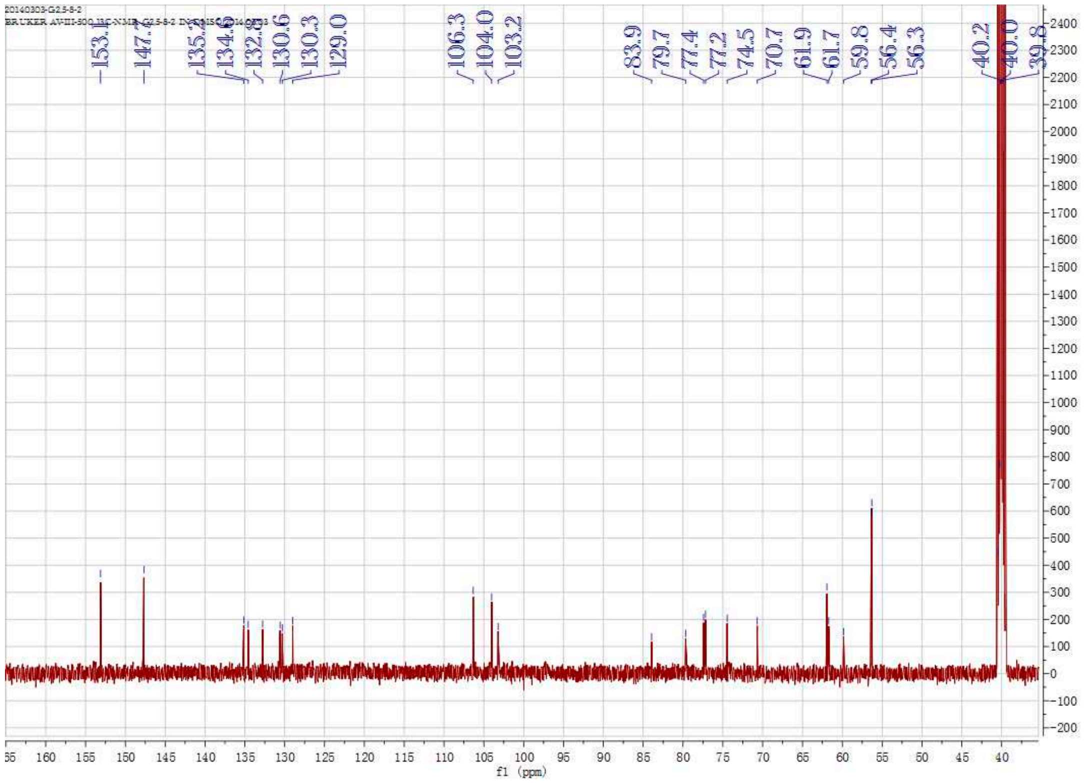

Figure S31. ^{13}C-NMR of compound **10** (Data acquired in DMSO-d_6 at 500 MHz).

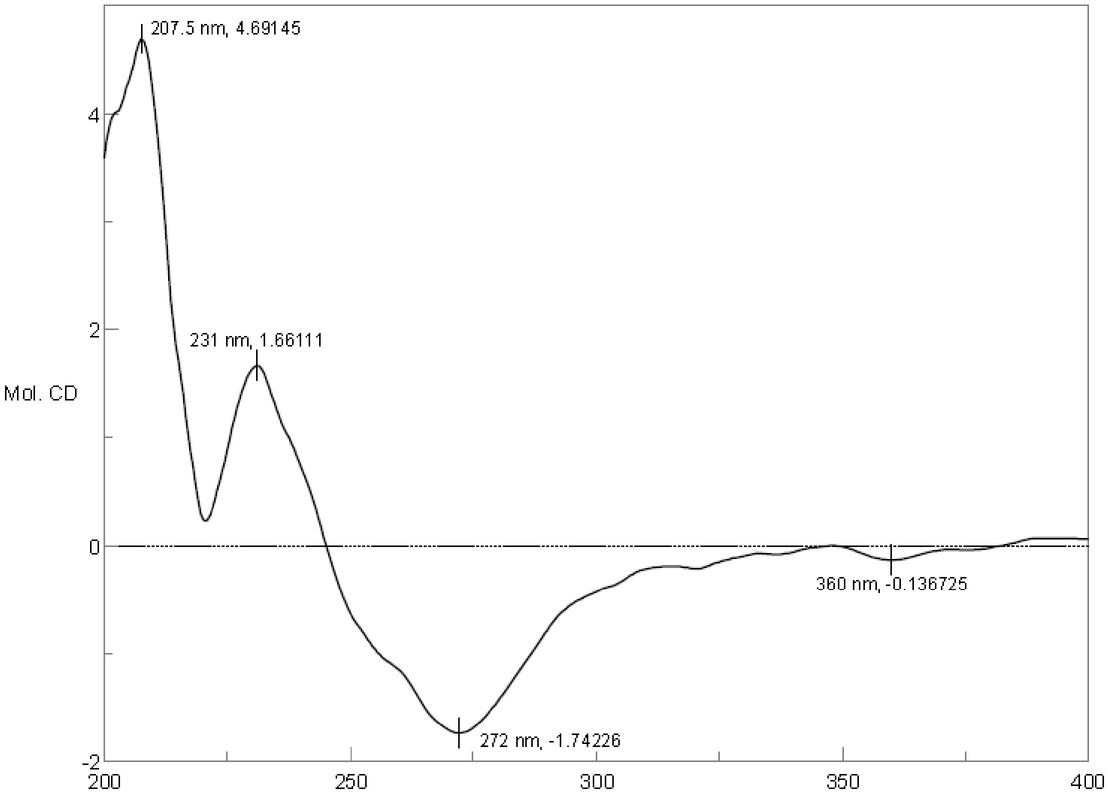

Figure S32. CD of compound **10**.
